# *Premna odorata* extract exhibits anti-inflammatory activity via suppression of NLRP3 inflammasome

**DOI:** 10.1038/s41598-026-55217-1

**Published:** 2026-06-10

**Authors:** Jaehyeok Lee, Seyoung Kim, Seongjong Lee, Hangyeol Lee, Hyeyun Yang, Seoyeon Jang, Sojin Yun, Ja Hyang Cho, Man S. Kim, Hyung Won Ryu, Jung Hee Kim, Dong-Keun Yi, Soo-Yong Kim, SangHo Choi, Sang Woo Lee, Md. Salah Uddin, Hyoung-Geun Kim, Yoonsung Lee, Yong Hwan Park

**Affiliations:** 1 BK21 R&E Initiative for Advanced Precision Medicine, Suwon, Republic of Korea; 2 Department of Biomedical Sciences, Graduate School of Ajou University, Suwon, 16499 Republic of Korea; 3 Department of Microbiology, Ajou University School of Medicine, Suwon, 16499 Republic of Korea; 4 Department of Biomedical Science and Technology, Kyung Hee University, Seoul, 05278 Republic of Korea; 5 Clinical Research Institute, Kyung Hee University Hospital at Gangdong, School of Medicine, Kyung Hee University, Seoul, 05278 Republic of Korea; 6 Department of Pediatrics, Kyung Hee University Hospital at Gangdong, College of Medicine, Kyung Hee University, Seoul, 05278 Republic of Korea; 7 Natural Product Research Center, Korea Research Institute of Bioscience and Biotechnology, Cheongju-si, 28116 Chungcheongbuk-do Republic of Korea; 8 International Biological Material Research Center, Korea Research Institute of Bioscience and Biotechnology, Daejeon, 34141 Republic of Korea; 9 Botanika, Botanical Research Centre, Tejgaon, Dhaka, 1208 Bangladesh; 10 G-LAMP NEXUS Institute, Kyung Hee University, Yongin, 17104 Republic of Korea

**Keywords:** Anti-inflammatory activity, Inflammation, NLRP3 inflammasome, Interleukin-1β, *Premna odorata*, Biochemistry, Drug discovery, Immunology, Microbiology

## Abstract

*Premna odorata* (family Verbenaceae), known as “Alagaw” in the Philippines, has traditionally been used to treat inflammatory respiratory conditions; however, molecular evidence supporting its pharmacological effects remains limited. Given that NOD-like receptor pyrin domain-containing protein 3 (NLRP3) inflammasome-driven IL-1β secretion plays a central role in inflammatory respiratory conditions, we investigated whether *P. odorata* extract (POE) modulates NLRP3 inflammasome activation. In LPS-primed J774A.1 and THP-1 macrophages, POE reduced IL-1β secretion induced by ATP or nigericin without affecting NLRC4 or AIM2 inflammasome activation, indicating NLRP3 selectivity. POE treatment inhibited adaptor apoptosis-associated speck-like protein containing CARD (ASC) oligomerization and speck formation without affecting cell viability or intracellular ROS levels, indicating suppression of inflammasome assembly. In vivo, POE attenuated inflammatory cell infiltration in an LPS-induced zebrafish model. Together, these findings indicate that POE suppresses NLRP3 inflammasome–mediated inflammatory responses, providing a molecular basis for its traditional use in managing inflammatory conditions and offering a promising natural scaffold for the development of NLRP3-targeted therapeutics.

**Supplementary Information:**

The online version contains supplementary material available at 10.1038/s41598-026-55217-1.

## Introduction

Dysregulated activation of the NLRP3 inflammasome has been implicated in various inflammatory diseases, including autoimmune disorders, asthma^1^, cryopyrin-associated periodic syndromes (CAPS)^2^, and neurodegenerative conditions^3^. Functionally, the NLRP3 inflammasome is a cytosolic multiprotein complex that facilitates the processing and secretion of IL-1β and IL-18, which amplifies pathological inflammation^4^. The NLRP3 inflammasome assembles in response to diverse cellular stress signals. Upon its activation, it recruits the adaptor apoptosis-associated speck-like protein containing CARD (ASC) and pro-caspase-1, leading to the formation of the inflammasome complex^5^. Activated caspase-1 cleaves pro-IL-1β and pro-IL-18 into their mature forms, IL-1β and IL-18, which are subsequently released, amplifying inflammatory responses^6^. Although this process is essential for host defense under physiological conditions, persistent or aberrant activation of the NLRP3 inflammasome contributes to pathological inflammation^5^ and tissue damage. Consequently, inhibition of NLRP3 inflammasome activation has been explored as a therapeutic strategy. NLRP3 inflammasome activation occurs through a two-step process: priming and activation^4,7^. During priming, ligands, such as LPS, bind to Toll-like receptor 4 (TLR4), activating NF-κB signaling and promoting the transcription of pro-IL-1β and NLRP3^8^. The subsequent activation step is triggered by intracellular stress signals, such as potassium efflux^9^, reactive oxygen species (ROS) production^10^, lysosomal rupture^11^, and mitochondrial dysfunction^12^. These signals induce conformational changes in NLRP3, thereby promoting its oligomerization and inflammasome assembly. Concurrently, the NLRP3 inflammasome cleaves gasdermin D, which forms membrane pores that facilitate the extracellular release of cytokines^13^. Given the involvement of NLRP3 in chronic inflammation, efforts to identify safe and selective NLRP3 inhibitors have gained momentum. Natural products with anti-inflammatory properties are of particular interest as inhibitors because of their structural diversity and historical uses in traditional medicine^14^. In this study, we examined the anti-inflammatory potential of *Premna odorata* extract (POE), a species belonging to the Verbenaceae family commonly known as “Alagaw” in the Philippines and is also widely distributed across Southeast Asia. POE has been used extensively in traditional Southeast Asian medicine for the treatment of cough^15^ and bronchitis^16^, primarily administered as a decoction of leaves, which has been traditionally consumed as herbal tea to alleviate phlegm and respiratory inflammation^16^. Phytochemical analyses have revealed that POE contains iridoids, flavonoids, and terpenoids as major constituents, which are known for their diverse pharmacological activities^17^, including antiviral and anti-inflammatory effects^18^. However, the molecular mechanisms through which POE exerts anti-inflammatory effects, particularly in the context of NLRP3 inflammasome regulation, remain unclear. Therefore, this study aimed to elucidate the molecular mechanisms by which POE inhibits NLRP3 inflammasome activation. Given its traditional use in inflammatory conditions, we hypothesized that POE may modulate inflammasome-mediated inflammation by interfering with NLRP3 complex formation. To test this hypothesis, we conducted in vitro experiments to determine whether POE suppresses NLRP3 inflammasome assembly. These effects were validated in vivo using a zebrafish inflammation model, which has the unique advantage of enabling real-time visualization of innate immune cells. Collectively, our findings indicate that POE attenuates NLRP3-mediated inflammatory responses in vitro and in vivo.

## Materials and methods

### Ethics statements

All experimental procedures were conducted in accordance with relevant institutional guidelines and regulations. Experiments involving zebrafish embryos were approved by the Institutional Animal Care and Use Committee (IACUC) of Kyung Hee University Hospital at Gangdong (Approval No. KHNMC AP 2024-009).

### Preparation of POE

The plant extract of *Premna odorata Blanco* (FBM297-033) used in this research was obtained from the International Biological Material Research Center at the Korea Research Institute of Bioscience and Biotechnology (Daejeon, Republic of Korea). The plant was collected from Hazarikhil district, Chittagong, Bangladesh in May 2016. The plant material was authenticated based on morphological characteristics by Dr. Uddin. A voucher specimen (KRIB 0075165) is kept in the herbarium of the Korea Research Institute of Bioscience and Biotechnology. The (100 g) dried leaves in the shade and powdered were added to 1 L of methyl alcohol 99.9% (HPLC grade) and extracted through 30 cycles (40 kHz, 1500 W, 15 min. ultrasonication-120 min. standing per cycle) at room temperature using an ultrasonic extractor (SDN-900 H, SD-ULTRASONIC CO., LTD). After filtration (Qualitative Filter No.100, HYUNDAI MICRO CO., LTD) and drying under reduced pressure, P. odorata extract (2 g) was obtained.

### Cell culture and stimulation

Human THP-1 and mouse J774A.1 cells (Korean Cell Line Bank, passage numbers between 5 and 15) were cultured according to ATCC guidelines. NLRP3-knockout (KO) THP-1 cells were maintained under the same culture conditions as THP-1 cells. An incubator set to 37 °C with 5% CO₂ was used to maintain the cells. THP-1 cells and NLRP3 KO THP-1 cells were differentiated with PMA (500 nM)^19–21^ for 3 h and used after an additional 2 days of incubation. J774A.1 cells were seeded at 0.2 × 10^6^ cells/well in 48-well plates 1 day prior to experimentation to ensure sufficient adhesion. To prime the cells, LPS (100 ng/mL) was added in a volume of 200 µL per well and incubated for 3 h. POE at (10, 50, or 100 µg/mL) was added without medium change, and incubation proceeded for another 2 h. The medium was then aspirated and replaced with 100 µL of ATP (5 mM), nigericin (10 µM) or imiquimod (200 µM) for 30 min to trigger inflammasome activation. dsDNA (2 µg/mL) and flagellin (1.25 µg/mL) were delivered following transfection with Lipofectamine 2000™ and incubated for 3 h. To activate the non-canonical inflammasome pathway, LPS (1.5 µg/mL) was transfected into cells using Lipidofect-P and incubated for 3 h, after which supernatants were collected. MCC950 (100 nM) was applied for 30 min as a positive inhibitory control.

### Cytotoxicity assay

Differentiated THP-1 cells and J774A.1 cells were plated at (0.5 × 10⁵ cells/well) in 96-well plates for cytotoxicity assays. THP-1 cells were exposed to POE (10, 50, or 100 µg/mL) and incubated overnight, while J774A.1 cells were incubated with POE at identical concentrations for 2 h. EZ-CYTOX was added for 2 h to assess cell viability. Absorbance was detected at 450 nm using an iMark™ reader (Bio-Rad, 168–1130).

### ELISA

DuoSet ELISA kits (R&D Systems, DY318-05, DY401-05) were employed to quantify IL-18 and IL-1β in human and mouse samples, following the manufacturer’s recommended procedures.

### Immunoblotting

Protein expression related to inflammasome activation was analyzed by immunoblotting. J774A.1 and THP-1 cells were seeded at 0.2 × 10⁶ cells/well in 48-well plates. Supernatants (50 µL) were collected from equal numbers of cells seeded under identical conditions and mixed with 5× SDS sample buffer (8 µL), followed by heating at 100 °C prior to SDS-PAGE. During heating, a slight reduction in volume occurred, resulting in a final volume of approximately 40 µL, which was fully loaded per lane. This approach was applied to improve the detectability of IL-1β under our experimental conditions.

Equal volumes of supernatants derived from equivalent cell numbers were used for all conditions, and no additional normalization step was applied. After supernatant collection, the remaining cells were washed with cold PBS and lysed in lysis buffer (Biological Industries, BI010) containing protease inhibitors. Cell lysates were cleared by centrifugation, mixed with SDS sample buffer, boiled, and subjected to SDS-PAGE. Lysates were analyzed in parallel to confirm comparable levels of pro–IL-1β and tubulin across samples, thereby validating equivalent cell numbers across conditions. Proteins were separated on 12% and 10% polyacrylamide gels for supernatant and cell lysate samples, respectively. For cell lysates, 25 µL was loaded per lane.

Membranes were incubated with primary antibodies overnight at 4 °C, followed by HRP-conjugated secondary antibodies for 1 h at room temperature. Bands were visualized using an ECL detection system. α-Tubulin expression in cell lysates was used to confirm equal loading across experimental groups. Detailed protocols for nuclear/cytoplasmic fractionation and ASC oligomerization are provided in the Supporting Information.

### NF-κB luciferase assay

HEK293FT cells (0.2 × 10^5^ cells/well) were plated into 96-well white plates and maintained for 24 h. The following day, transfection was performed with the pGL4.32 luciferase reporter plasmid (0.1 µg/well) using Lipofectamine 2000™, after which the cells were incubated for 24 h. Subsequently, cells were treated with TNF-α (20 ng/mL) for 3 h, followed by treatment with POE (10, 50, or 100 µg/mL) for an additional 2 h. Luciferase activity was assessed with the Bright-Glo™ Luciferase Assay System (Promega) and quantified with a BMG LABTECH FLUOstar OPTIMA reader.

### Immunofluorescence

ASC speck formation, mitochondrial ROS accumulation, and mitochondrial membrane potential were visualized by immunofluorescence staining and confocal microscopy. Detailed staining procedures are provided in the Supporting Information.

### NLRP3 ATPase activity assay

To assess NLRP3 ATPase function, recombinant human NLRP3 protein was treated with POE or control and analyzed using the ADP-Glo™ Max assay, as recommended by manufacturer’s instructions. Detailed procedures are provided in the Supporting Information.

### Zebrafish husbandry and ethics

Adult wild-type (WT) TAB5 zebrafish were obtained from the Korea Zebrafish Resource Center (KZRC, Republic of Korea) and maintained under standard laboratory conditions. Embryos were generated by natural mating and maintained in embryo medium containing N-phenylthiourea (PTU) at 28 °C. Embryos were dechorionated using pronase.

### Sudan Black B staining

At 3 dpf, embryos were stained by Sudan Black B staining and the caudal hematopoietic tissue (CHT) region was imaged using a Leica M250 C microscope, with details provided in the Supporting Information.

### In vivo imaging of transgenic zebrafish

Transgenic Tg *(mpeg1*: EGFP*)* zebrafish were crossed with WT zebrafish to obtain embryos. At 3 dpf, live embryos were anesthetized with tricaine and imaged in the CHT region using an Olympus FV3000RS confocal microscope, with details provided in the Supporting Information.

### UPLC–PDA-QTOF–MS conditions

Metabolite profiling was conducted using an ACQUITY UPLC system coupled to a Xevo G2-S QTOF mass spectrometer. Chromatographic separation was performed on a BEH C18 column under a formic acid–based water/acetonitrile gradient, and spectra were acquired in MSE mode with leucine-enkephalin for real-time mass correction. Additional instrumental parameters and detailed analytical conditions are provided in the Supporting Information.

### Statistical analysis

Data is presented as mean ± SEM. Statistical significance among multiple groups was determined using one-way analysis of variance (ANOVA) followed by Tukey’s post hoc test. Comparisons between two groups were performed using an unpaired Student’s t-test. All statistical analyses were conducted using GraphPad Prism version 8 (GraphPad Software, San Diego, CA, USA). A p value < 0.05 was considered statistically significant.

## Results

### POE attenuated IL-1β secretion mediated by NLRP3 activation

**Fig. 1:**
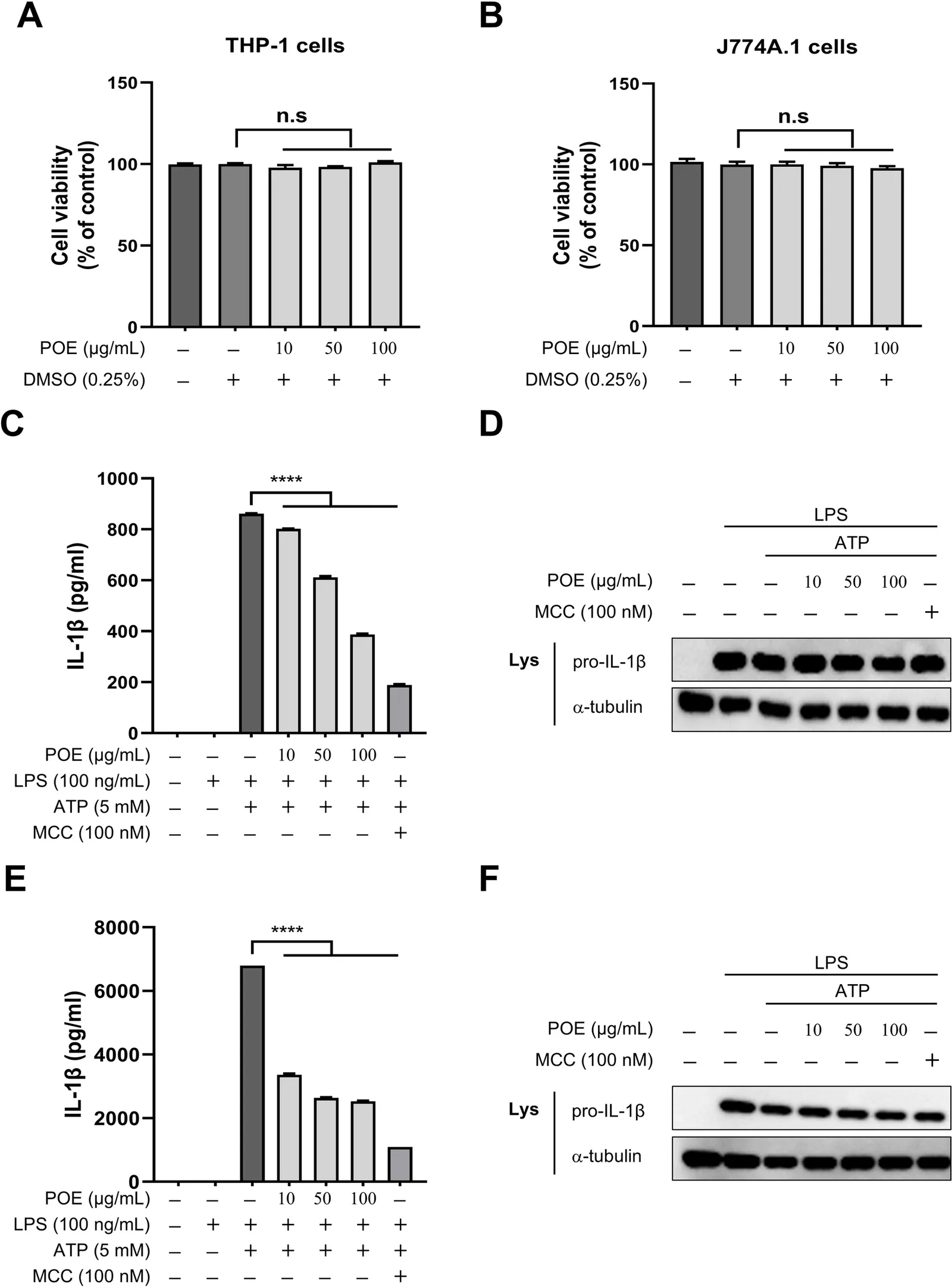
POE attenuated IL-1β secretion mediated by NLRP3 activation. (**A**) Differentiated THP-1 cells were treated with POE (10, 50, 100 µg/mL) overnight. (**B**) J774A.1 cells were treated with POE (10, 50, 100 µg/mL) for 2 h, and cell viability was measured using the EZ-CYTOX assay at 450 nm. Data are presented as mean ± SEM (*n* = 10). (**C**) THP-1 cells were primed with LPS (100 ng/mL) for 3 h, followed by treatment with POE for 2 h, and then stimulated with ATP (5 mM) for 30 min. IL-1β levels in the supernatant (Sup) were measured by ELISA. Data are presented as mean ± SEM (*n* = 3). (**D**) Soluble lysates (Lys) from LPS-primed THP-1 cells were analyzed by Western blot to detect pro-IL-1β and α-tubulin. (**E**) J774A.1 cells were primed with LPS (100 ng/mL) for 3 h, then treated with POE for 2 h, followed by ATP (5 mM) for 30 min. IL-1β levels in the supernatant (Sup) were measured by ELISA. Data are presented as mean ± SEM (*n* = 3). (**F**) Soluble lysates (Lys) from LPS-primed J774A.1 cells were analyzed by Western blot to detect pro-IL-1β and α-tubulin. Data are presented as mean ± SEM. Statistical significance was determined using one-way ANOVA followed by Tukey’s post hoc test. *p < 0.05; **p < 0.01; ***p < 0.001; ****p < 0.0001; n.s., not significant. For each panel, all samples were run on the same gel and processed under identical experimental conditions. Blots were cropped for clarity; full-length blots are provided in the Supplementary Information.

Treatment with various concentrations of POE (10, 50, or 100 µg/mL) did not affect cell viability in either THP-1 or J774A.1 cells when applied for 24 and 2 h, respectively (Fig. 1A,B). Cytotoxicity was observed at concentrations exceeding 100 µg/mL in both cell lines (Supplementary Fig. 1), and thus 100 µg/mL was set as the maximum concentration for subsequent experiments. To assess the potential of POE to inhibit the NLRP3 inflammasome, THP-1 macrophages were primed with LPS and subsequently treated with increasing concentrations of POE, followed by stimulation with ATP, which induces potassium efflux via P2X7 receptor activation to trigger NLRP3 inflammasome assembly. POE decreased IL-1β secretion in a concentration-dependent manner in LPS-primed THP-1 macrophages (Fig. 1C). Notably, POE reduced IL-1β secretion to an extent comparable to that observed with MCC950 under these experimental conditions^22^, a well-characterized and selective NLRP3 inhibitor. Meanwhile, α-tubulin and pro-IL-1β levels remained unchanged, indicating that POE does not nonspecifically suppress protein synthesis or induce cytotoxicity (Fig. 1D). Consistent results were obtained in the murine macrophage cell line J774A.1, in which higher concentrations of POE also suppressed IL-1β release without affecting α-tubulin or pro-IL-1β expression (Fig. 1E,F). Furthermore, IL-18, another key cytokine released upon NLRP3 inflammasome activation, was also reduced by POE treatment in a concentration-dependent manner in LPS-primed J774A.1 cells stimulated with ATP (Supplementary Fig. 2). Collectively, these findings indicated that POE attenuated IL-1β secretion induced by NLRP3 activation in both human and murine macrophages, and that the extent of IL-1β reduction was comparable to that observed with MCC950.

### POE inhibited NLRP3 inflammasome activation without affecting NF-κB signaling or other inflammasomes

**Fig. 2:**
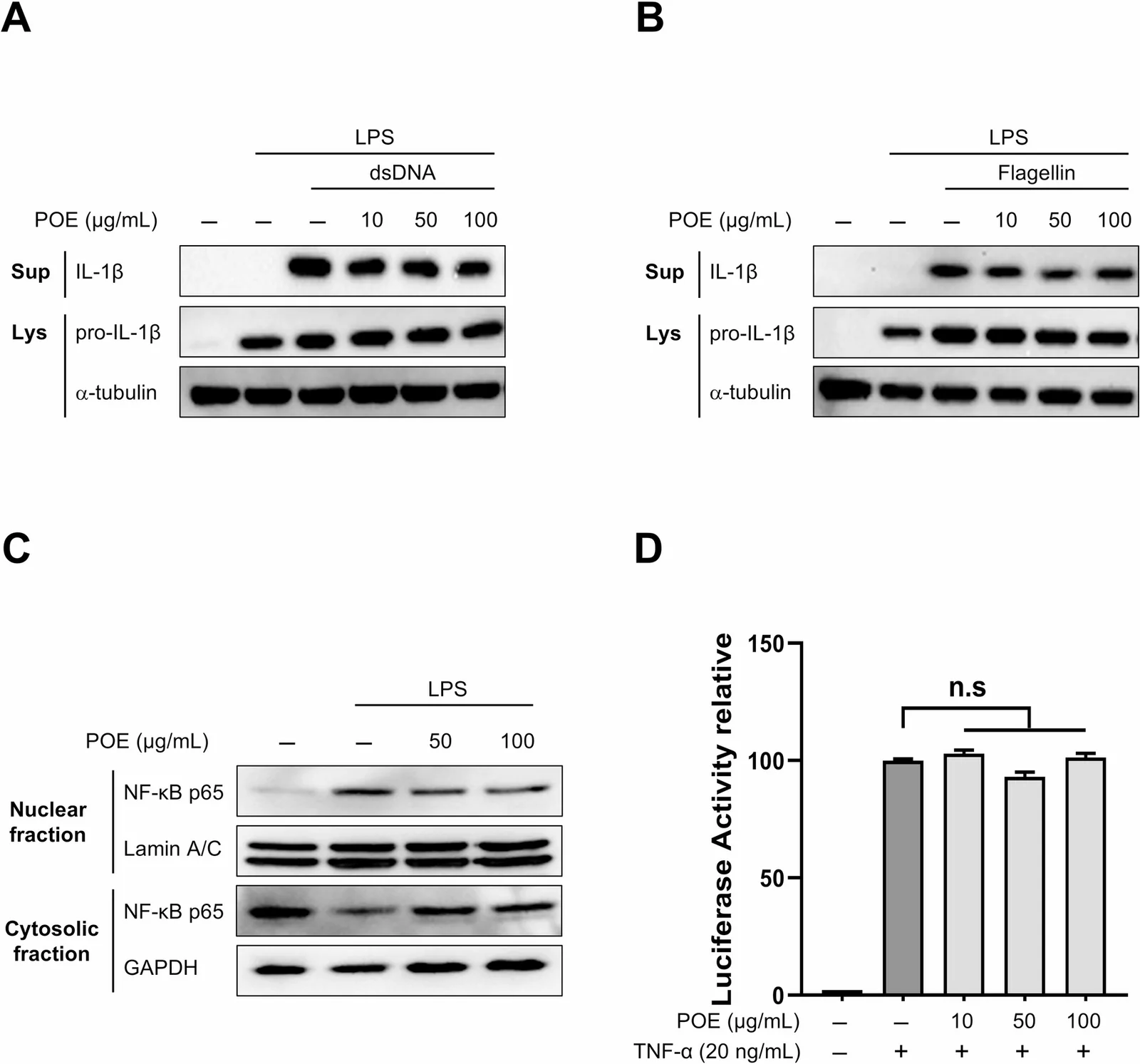
POE inhibited NLRP3 inflammasome activation without affecting NF-kB signaling or other inflammasomes. (**A**, **B**) J774A.1 cells were treated with POE and transfected with (**A**) dsDNA (2 µg/mL) or (**B**) flagellin (1.25 µg/mL) using Lipofectamine 2000 to activate the AIM2 and NLRC4 inflammasomes. (**C**) LPS-primed J774A.1 cells were treated with POE for 2 h, and nuclear and cytosolic fractions were prepared, and NF-κB p65 translocation was analyzed by immunoblotting. (**D**) 293 FT cells were transfected with the NF-kB luciferase reporter vector (pGL4.32), stimulated with TNF-α (20 ng/mL) in the presence or absence of POE for 5 h, and luciferase activity was measured using the Promega Bright-Glo Luciferase Assay System. Data are presented as mean ± SEM (*n* = 5). Statistical significance was determined using one-way ANOVA followed by Tukey’s post hoc test. *p < 0.05; **p < 0.01; ***p < 0.001; ****p < 0.0001; n.s., not significant. For each panel, all samples were run on the same gel and processed under identical experimental conditions. Blots were cropped for clarity; full-length blots are provided in the Supplementary Information.

To determine whether POE affects inflammasomes other than NLRP3, we stimulated cells with known activators of the AIM2 (dsDNA) and NLRC4 (flagellin) inflammasomes in the presence of POE (Fig. 2A,B). IL-1β secretion levels remained unchanged across all POE concentrations, suggesting that POE preferentially inhibited the NLRP3 inflammasome. NLRP3 activation is governed by a two-step process: priming and activation. Therefore, we investigated whether POE interfered with the priming step. To this end, NF-κB signaling was induced in J774A.1 cells using LPS, followed by POE treatment. Nuclear translocation of p65, a key NF-κB subunit, showed no discernible changes compared with that in the untreated controls (Fig. 2C). To further validate this finding, an NF-κB luciferase reporter assay was performed. POE treatment had no significant effect on NF-κB transcriptional activity or expression (Fig. 2D). Collectively, these results indicated that POE selectively inhibited NLRP3 inflammasome activation without perturbing NF-κB signaling or activating other inflammasomes.

### POE inhibited canonical NLRP3 activation without affecting K⁺ efflux or ROS and suppresses noncanonical activation

**Fig. 3:**
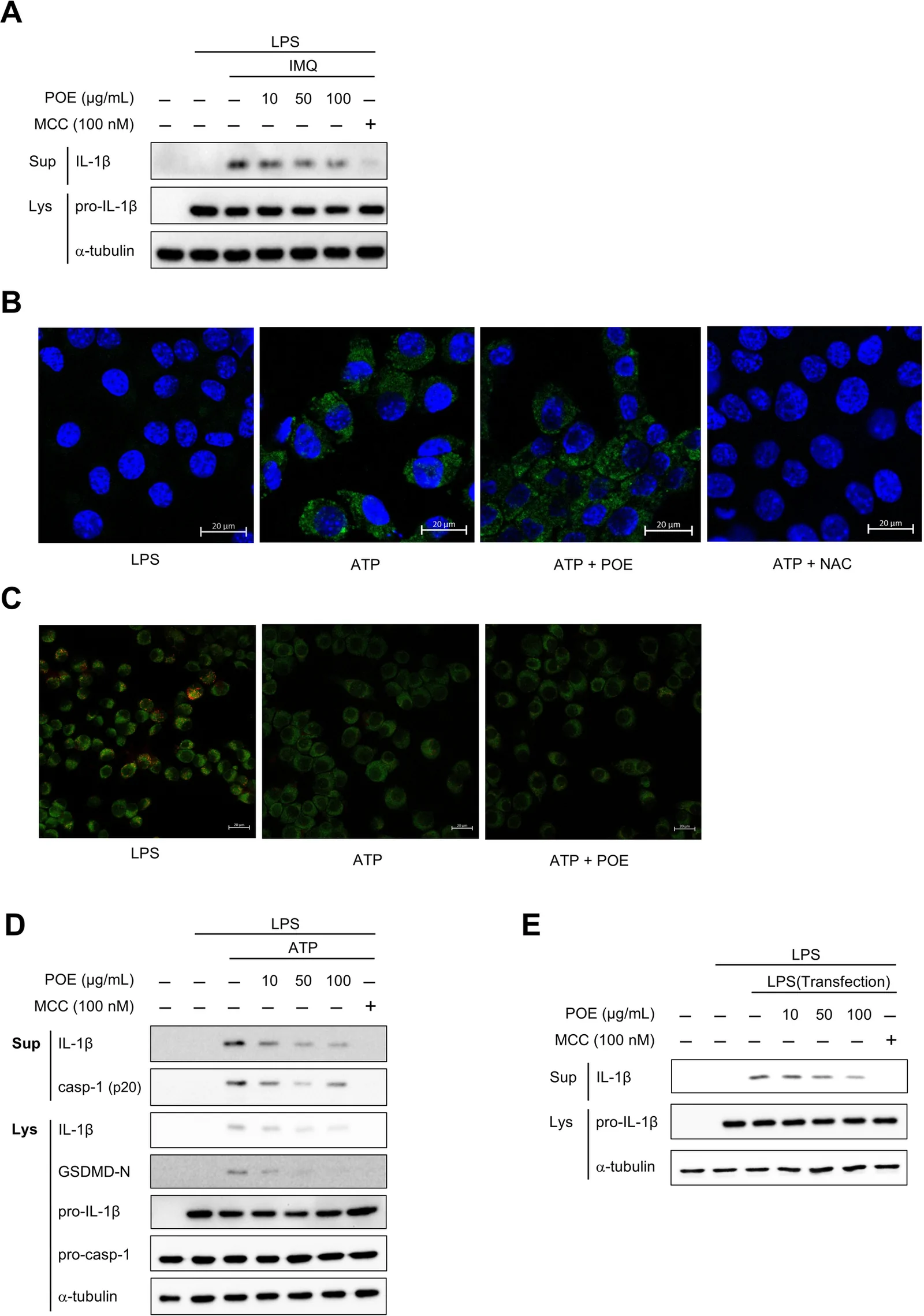
POE inhibits canonical NLRP3 activation without affecting K⁺ efflux or ROS and suppresses noncanonical activation. (**A**) LPS-primed J774A.1 cells were treated with POE for 2 h, followed by stimulation with imiquimod (IMQ, 200 µM) for 30 min to activate the NLRP3 inflammasome. (**B**) LPS (100 ng/mL)-primed J774A.1 cells were treated with POE (50 µg/mL) for 2 h. NAC (3 mM), an intracellular ROS scavenger, was co-treated with LPS for a total of 5 h and used as a negative control. Cell was then stimulated with ATP (5 mM) for 5 min. Intracellular ROS levels were visualized by confocal laser-scanning microscopy (Carl Zeiss LSM710). Scale bar: 20 μm. (**C**) LPS (100 ng/mL)-primed J774A.1 cells were treated with POE (50 µg/mL) for 2 h, followed by incubation with JC-1 dye (10 µM) for 10 min. Cells were then stimulated with ATP (5 mM) for 5 min. Mitochondrial membrane potential was assessed by confocal laser-scanning microscopy (Carl Zeiss LSM710). Scale bar: 20 μm. (**D**) LPS-primed J774A.1 cells were treated with POE (10, 50, or 100 µg/mL) for 2 h and stimulated with ATP (5 mM) for 30 min. Cleaved caspase-1 (p20), GSDMD-N, IL-1β, pro-IL-1β, pro-caspase-1, and α-tubulin were analyzed by Western blot. (**E**) J774A.1 cells were primed with LPS (100 ng/mL) for 3 h, followed by POE (10, 50, or 100 µg/mL) treatment for 2 h. LPS (1.5 µg/mL) was then transfected into cells for 3 h to activate the non-canonical inflammasome pathway. IL-1β in the supernatant and pro-IL-1β and α-tubulin in the cell lysate were analyzed by Western blot. For each panel, all samples were run on the same gel and processed under identical experimental conditions. Blots were cropped for clarity; full-length blots are provided in the Supplementary Information.

Activation of the NLRP3 inflammasome is commonly initiated by intracellular stress signals such as potassium (K⁺) efflux and ROS accumulation. To determine whether the inhibitory effect of POE is mediated through these pathways, we first tested its activity in a K⁺ efflux–independent activation model. J774A.1 cells were stimulated with imiquimod (IMQ), an NLRP3 activator known to bypass the requirement for K⁺ efflux, in the presence of POE. As shown in (Fig. 3A), POE treatment significantly reduced IL-1β secretion, suggesting that its inhibitory effect is unlikely to be mediated by inhibition of K⁺ efflux under these conditions. Next, we examined whether POE modulates ROS accumulation. Intracellular ROS levels increased following ATP stimulation but remained unchanged following POE treatment, whereas ROS accumulation was markedly reduced following administration of the ROS scavenger N-acetylcysteine (NAC) (Fig. 3B). These findings suggest that POE does not reduce intracellular ROS levels under the tested conditions. Furthermore, we assessed whether POE affects mitochondrial membrane potential (ΔΨm) using the JC-1 fluorescent dye. Under normal conditions, JC-1 accumulates in polarized mitochondria as red-fluorescent J-aggregates. Upon mitochondrial depolarization, the red fluorescence signal diminishes as JC-1 dissociates from the mitochondria. ATP stimulation induced a marked reduction in red fluorescence, indicating mitochondrial membrane depolarization. This depolarization was not recovered by POE treatment (Fig. 3C), demonstrating that POE does not suppress NLRP3 inflammasome activation through restoration of mitochondrial membrane potential. Despite the independence of POE’s inhibitory effects from these upstream stress signals, POE reduced the levels of cleaved caspase-1 (p20) and the gasdermin D N-terminal fragment (GSDMD-N) in LPS-primed J774A.1 cells stimulated with ATP (Fig. 3D) in a concentration-dependent manner, indicating suppression of NLRP3 inflammasome activation and downstream pyroptotic signaling. Furthermore, to determine whether this inhibitory activity also extends to the non-canonical inflammasome pathway, LPS was transfected into J774A.1 cells. As shown in (Fig. 3E), POE treatment reduced IL-1β secretion in the supernatant in a concentration-dependent manner, supporting the possibility that POE acts on a shared downstream node of both pathways, most likely NLRP3 itself.

### POE inhibited NLRP3 inflammasome assembly and reduced NLRP3 ATPase activity

**Fig. 4:**
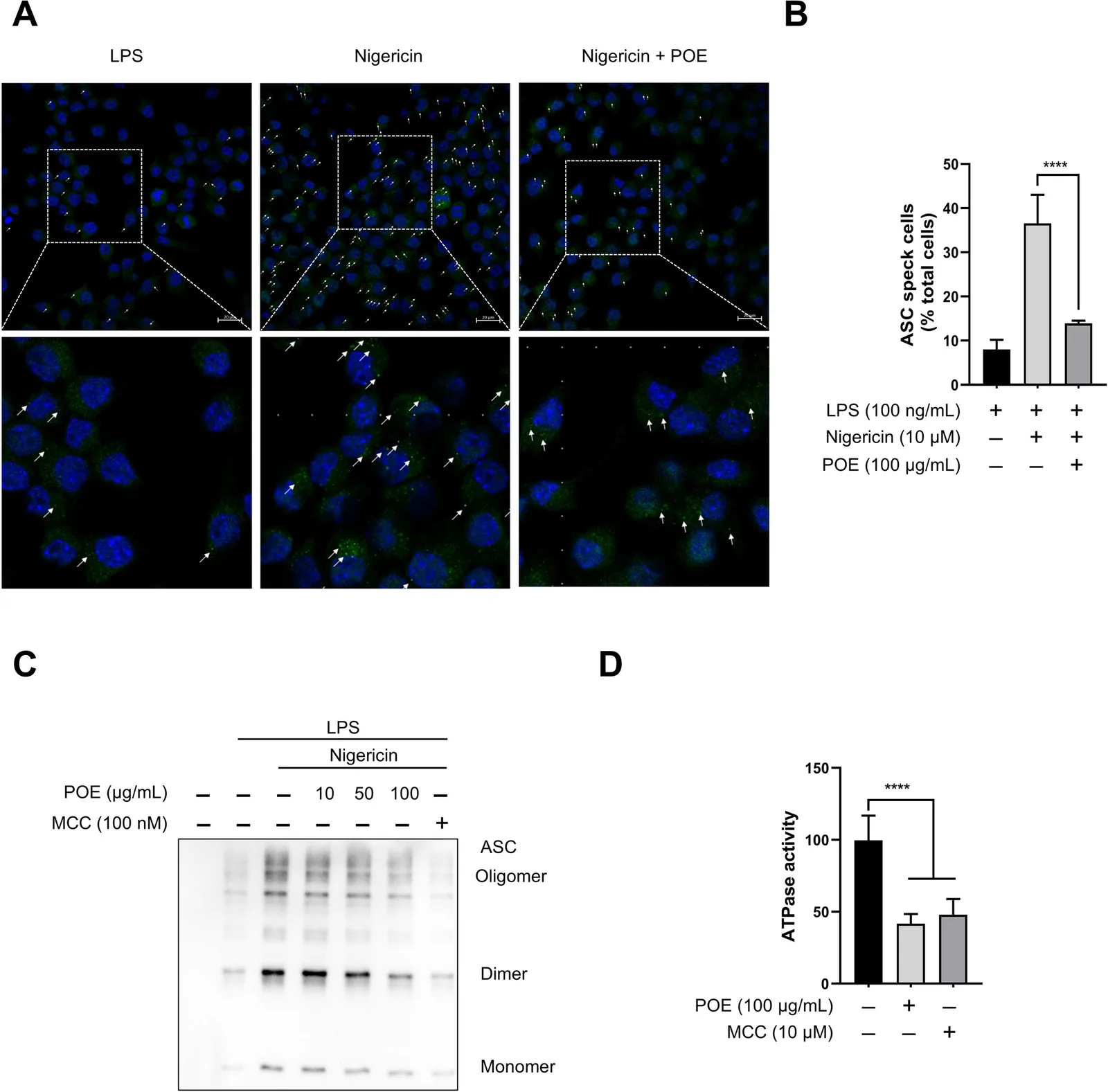
POE inhibited NLRP3 inflammasome assembly by inhibiting the ATPase activity of NLRP3. (**A**) LPS-primed J774A.1 cells were treated with POE (100 µg/mL) for 2 h and subsequently stimulated with nigericin (10 µM) for 1 h. Representative immunofluorescence images show ASC speck formation (Carl Zeiss, LSM710). (**B**) Quantification of ASC specks was performed based on immunofluorescence images. (**C**) Differentiated THP-1 cell pellets were cross-linked using disuccinimidyl suberate (DSS, 25 mM), and ASC oligomerization was assessed by Western blot. (**D**) ATPase activity of NLRP3 following POE treatment was measured using the ADP-Glo™ luminescence assay. Data are presented as mean ± SEM (*n* = 6). Statistical significance was determined using one-way ANOVA followed by Tukey’s post hoc test. *p < 0.05; **p < 0.01; ***p < 0.001; ****p < 0.0001; n.s., not significant. For each panel, all samples were run on the same gel and processed under identical experimental conditions. Blots were cropped for clarity; full-length blots are provided in the Supplementary Information.

Based on the observation that POE suppresses NLRP3 inflammasome activation independently of classical inhibitory signals, such as K⁺ efflux and ROS accumulation, we next investigated whether POE interferes with inflammasome formation. LPS-primed J774A.1 cells were stimulated with nigericin to activate the NLRP3 inflammasome and then treated with POE. Confocal microscopy revealed a significant reduction in ASC speck formation in POE-treated cells (Fig. 4A,B), suggesting that POE impaired inflammasome assembly. Consistently, POE inhibited ASC oligomerization in differentiated THP-1 cells in a concentration-dependent manner, with the highest concentration exhibiting effects similar to those observed with MCC950 under these conditions (Fig. 4C). To determine whether this inhibitory effect reflects a direct action on ASC or is mediated through NLRP3, ASC oligomerization was assessed in NLRP3-knockout (KO) THP-1 cells. In NLRP3-KO cells, neither ASC oligomer nor dimer formation was observed under any treatment condition, and ASC monomer levels remained consistent regardless of POE concentration (Supplementary Fig. 3). These results indicate that POE does not directly affect ASC itself, and that the suppression of ASC oligomerization is NLRP3-dependent. Notably, the ATPase activity of NLRP3 is essential for its activation, and it can be targeted by small-molecule inhibitors. To determine whether POE acts *via* this mechanism, we assessed the ATPase activity of NLRP3 after ATP stimulation. As shown in (Fig. 4D), POE significantly inhibited NLRP3 ATPase activity at a level comparable to that of MCC950. Taken together, these results indicated that POE suppressed NLRP3 inflammasome activation by disrupting ASC oligomerization and inhibiting the ATPase activity of NLRP3.

### The in vivo safety and anti-inflammatory activities of POE were evaluated using zebrafish

Based on the cellular experimental results, we examined the effects of POE in vivo using a zebrafish model. Zebrafish offers key advantages as a vertebrate model system, including rapid development, low maintenance costs, and a conserved immune system that closely resembles that of humans^23,24^. Moreover, their transparent embryos allow real-time visualization of innate immune cells, such as neutrophils and macrophages.

**Fig. 5:**
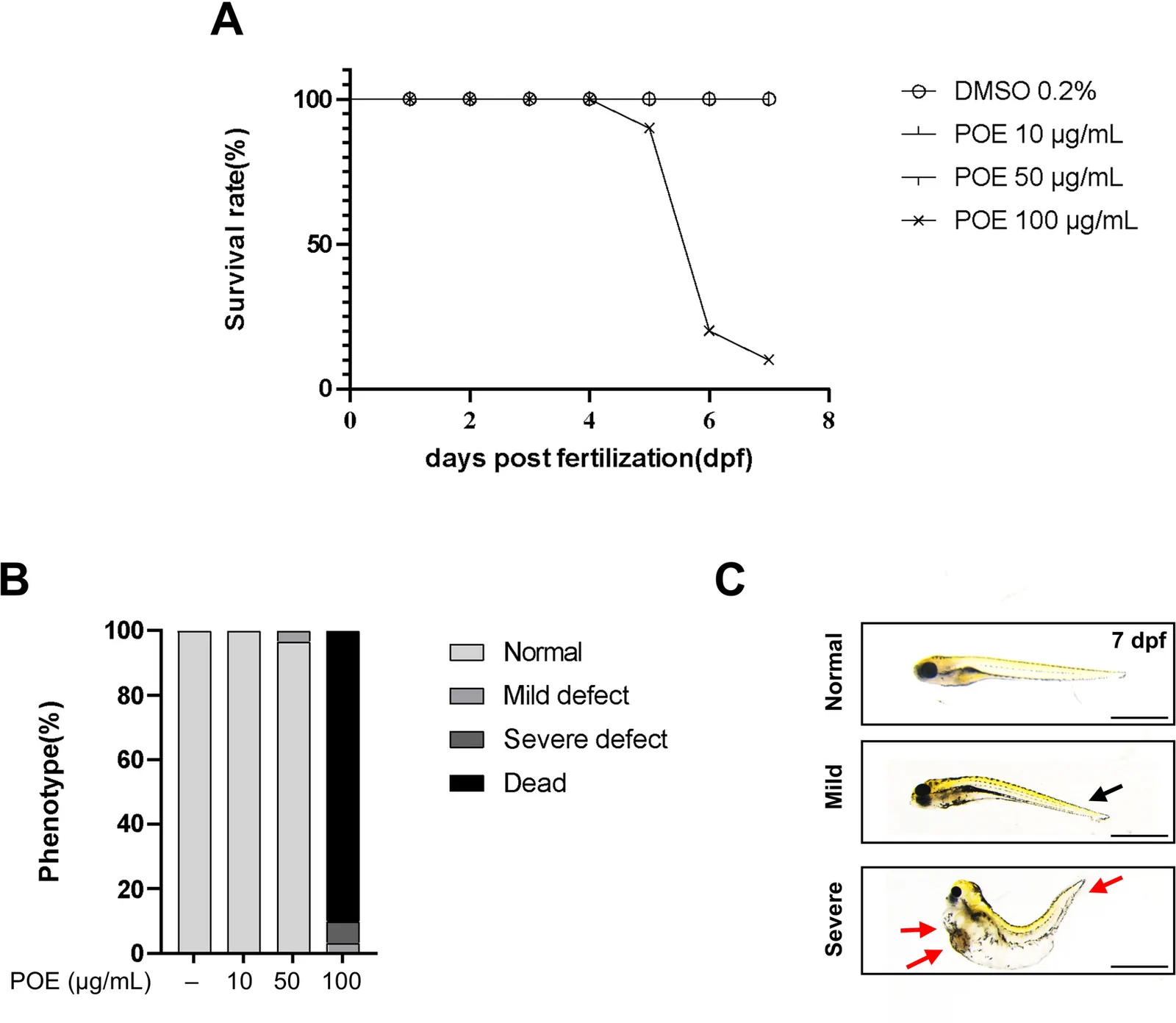
POE causes lethality and developmental toxicity at high concentrations but shows no adverse effects at lower doses in zebrafish embryos. (**A**) Survival curves of zebrafish embryos treated with POE (10, 50, 100 µg/mL) from 1 to 7 dpf. Thirty embryos per group (*n* = 30) were analyzed in a single independent experiment. Embryos were maintained in E3 medium, which was replaced daily. (**B**) Phenotypic distribution of surviving embryos at 7 dpf under different concentrations. (**C**) Representative images of the 7 dpf embryos corresponding to (**B**). Upper panel: control embryos treated with 0.2% DMSO in E3. Middle panel: mild defects, including spinal curvature (black arrow). Lower panel: severe defects included edema, abnormal yolk extension, and spinal curvature (red arrows). Scale bar, 1 mm.

To assess the potential toxicity, embryos were treated with POE at concentrations of (10, 50, and 100 µg/mL) from 1 to 7 days post-fertilization (dpf). POE at (10 and 50 µg/mL) was not lethal, whereas 100 µg/mL POE caused lethality (Fig. 5A). Embryos exposed to 100 µg/mL POE displayed severe developmental abnormalities, including edemas, failure of somite and brain formation, and spinal curvature defects (Fig. 5B,C), consistent with reported toxicity phenotypes^25^. In contrast, all embryos in the 10 and 50 µg/mL groups survived and showed no major developmental abnormalities. These results indicate that larval development is sustained at concentrations of POE below 50 µg/mL.

**Fig. 6:**
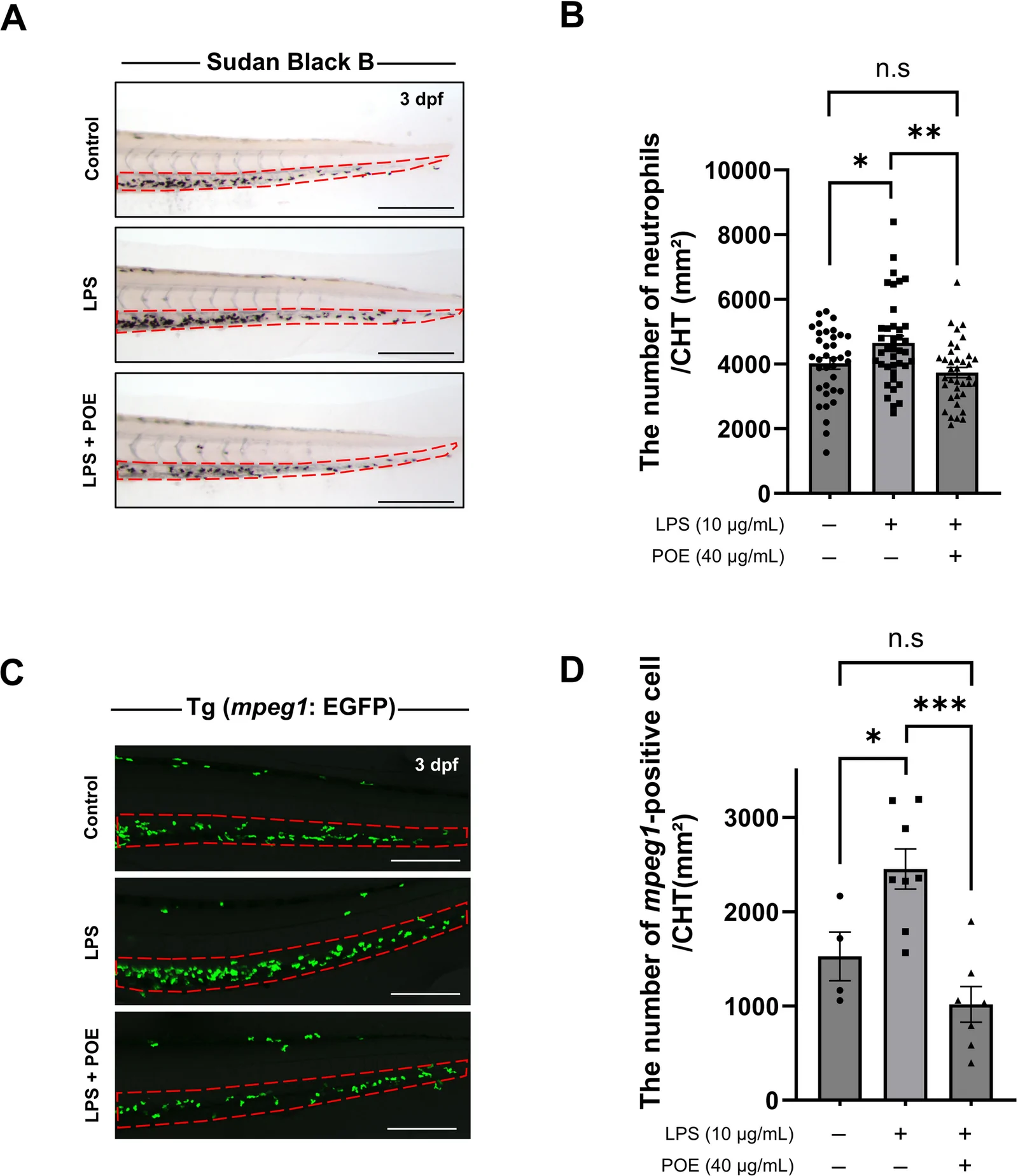
POE exhibits anti-inflammatory effects in zebrafish embryos. (**A**) Representative bright-field images of Sudan Black B staining in 3 dpf zebrafish embryos showing neutrophils in caudal hematopoietic tissue (CHT, outlined by red dashed line). (**B**) Quantification of neutrophils within the CHT corresponding to (**A**) from two independent biological replicate experiments. The number of embryos: Control (*n* = 36), LPS (*n* = 39), and LPS + POE (*n* = 37). (**C**) Representative confocal images of macrophage-positive cells in the CHT of Tg (*mpeg1*: EGFP) 3 dpf embryos (outlined by red dashed line). (**D**) Quantification of *mpeg1*-positive cells within the CHT corresponding to (**C**) from one independent biological experiment. The number of embryos: Control (*n* = 4), LPS (*n* = 8), and LPS + POE (*n* = 7). Control groups were treated with 0.08% DMSO, LPS groups were treated with LPS (10 µg/mL) to induce inflammation, and LPS + POE groups were co-treated with LPS (10 µg/mL) and POE (40 µg/mL) for 48 h from 1 dpf to 3 dpf. Data are presented as mean ± SEM in each graph. The p values were calculated using one-way ANOVA followed by Tukey’s post hoc HSD. ***p < 0.001; **p ≤ 0.01; *p < 0.05; and n.s., not significantly different. Scale bar, 300 μm.

To evaluate the anti-inflammatory effects of POE within the safe concentration range (10–50 µg/mL), we used a zebrafish inflammation model. As in previous studies, inflammation was induced *via* LPS stimulation, and neutrophil and macrophage recruitment in the CHT was assessed. Neutrophils initiate the immune response, followed by phagocytosis by macrophages in zebrafish embryos^26,27^. Sudan Black B–positive cells are generally considered to represent neutrophils, as this staining method is widely used to visualize neutrophils at early larval stages (e.g., 3 dpf) in zebrafish^28,29^. At 40 µg/mL POE, Sudan Black B staining revealed a significant reduction in neutrophil numbers (Fig. 6A,B), whereas 10 µg/mL POE did not alter the quantity of neutrophils (Supplementary Fig. 4). Furthermore, using Tg *(mpeg1*: EGFP*)* embryos, we found that macrophage recruitment under LPS stimulation decreased following 40 µg/mL POE treatment (Fig. 6C,D). These results demonstrated that POE suppressed LPS-induced neutrophil and macrophage responses in zebrafish embryos.

### UPLC-QTOF-MS analysis reveals the metabolite profile of POE

**Fig. 7:**
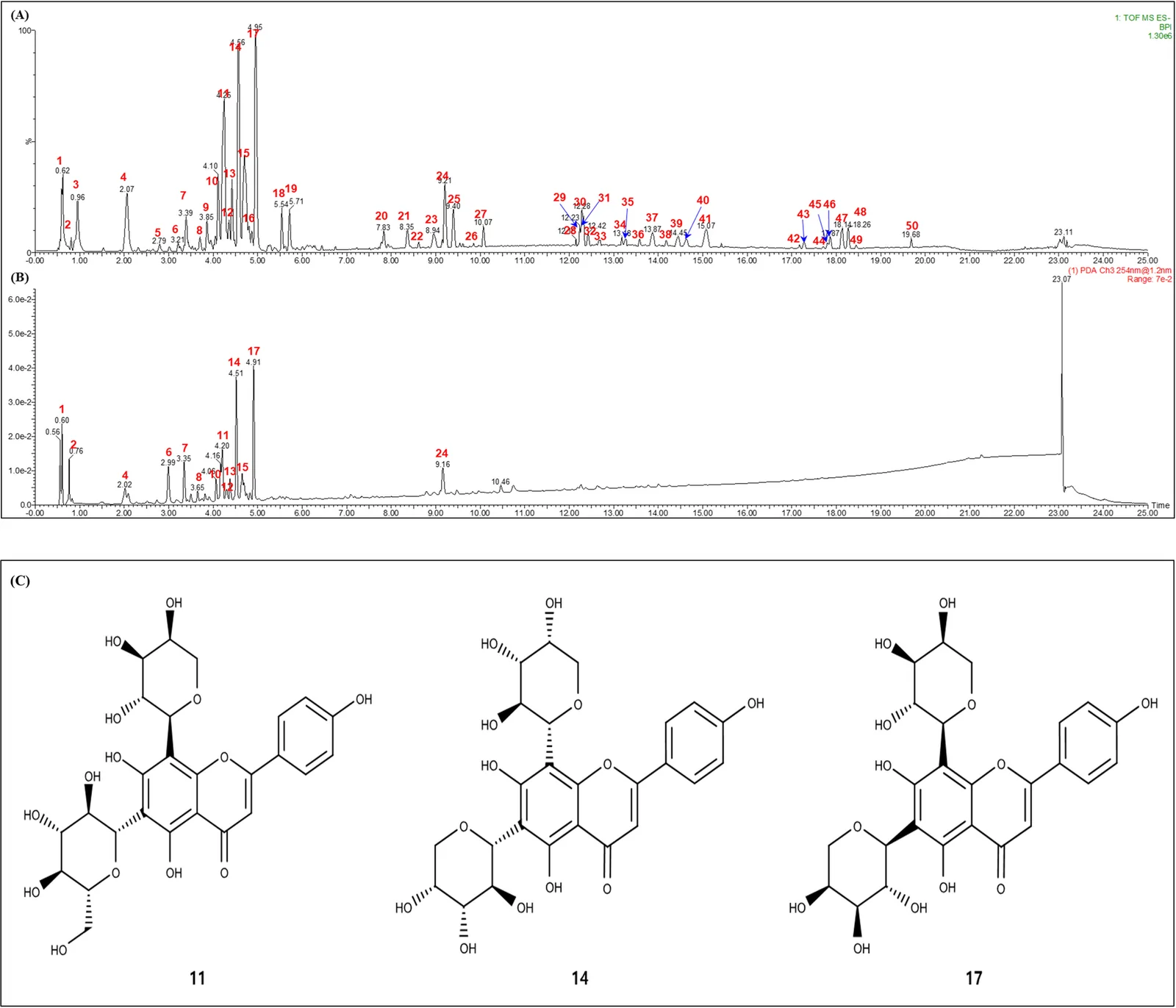
UPLC-QTOF-MS analysis reveals the metabolite profile of POE. (**A**) Annotated base peak ion (BPI) and (**B**) PDA (254 nm) chromatograms of the methanolic extract of *Premna odorata* analyzed by UPLC–QTOF–MS in the negative ionization mode. Peaks 1–50 correspond to metabolites that were tentatively identified and listed in Table S1. (**C**) The structure of the main peaks of POE.

Fifty metabolites were tentatively identified from the methanolic extract of POE using UPLC–QTOF–MS in negative ionization mode. Metabolites were identified based on accurate mass, retention time, and MS/MS fragmentation patterns. These compounds were classified into eight chemical categories: carboxylic acid derivatives, flavonoids, phenolic compounds, monoterpenes, fatty acids and esters, glycosides, triterpenes, and miscellaneous metabolites (Fig. 7A).

Based on the relative abundances observed in the PDA chromatograms at 254 nm (Fig. 7B), flavonoids were identified as the predominant group of metabolites. Among them, compounds **11** (area % = 5.32), **14** (area % = 11.78), and **17** (area % = 12.80) exhibited the highest relative abundances (Fig. 7C). This was followed by phenolic compounds **6** (area % = 5.29), **7** (area % = 4.32), and **24** (area % = 4.82), which were present at intermediate levels. In contrast, other metabolites, such as fatty acids, carboxylic acids, glycosides, and triterpenoids, were detected at comparatively low concentrations. References supporting the tentative identification, diagnostic ions, and neutral losses of the 50 metabolites are summarized in Table S1.

## Discussion

Research on the therapeutic potential of natural products for modulating inflammatory pathways has garnered increasing attention, particularly in the context of targeting the NLRP3 inflammasome, which has been implicated in chronic inflammation and a broad spectrum of diseases. In this study, we show that POE suppresses NLRP3 inflammasome activation. Notably, POE exerted selective anti-inflammatory effects without perturbing the NF-κB signaling pathway or interfering with other inflammasome subtypes, such as AIM2 or NLRC4. This selectivity may reduce the risk of generalized immunosuppression associated with broad-spectrum anti-inflammatory agents. Importantly, POE inhibited the assembly of the NLRP3 inflammasome complex and reduced NLRP3 ATPase activity, a biochemical feature required for its activation^14,30,31^. These findings suggest that POE may act through mechanisms beyond upstream signaling modulation. These cellular findings were supported by our experiments using a zebrafish inflammation model, in which POE (40 µg/mL) markedly reduced LPS-induced neutrophil and macrophage recruitment. These in vivo results support the anti-inflammatory activity of POE at the organismal level. However, since the zebrafish model used in this study does not directly assess NLRP3-specific inflammasome activation, the proposed NLRP3-related mechanism is primarily supported by our in vitro findings, and further in vivo studies will be required to directly validate this mechanism. Given the chemical complexity of plant extracts, future studies should focus on the identification and structural characterization of the specific bioactive constituents responsible for these effects. POE contains iridoids and various secondary anti-inflammatory metabolites^18^. Our phytochemical profiling further revealed that POE is rich in flavonoid compounds with known anti-inflammatory activities. Importantly, by demonstrating that POE inhibits NLRP3 inflammasome activation, this study narrows the search pool for natural NLRP3-targeting compounds to the characterized constituents of POE identified by UPLC-QTOF-MS profiling. These constituents may collectively contribute to the NLRP3 inflammasome inhibition observed in our study, although their individual roles remain to be clarified. Whether the observed NLRP3 inhibition was attributable to a single active compound or to the combined effects of multiple components remains to be determined. The present findings provide a focused chemical framework for future activity-guided fractionation and single-compound validation studies. Approaches such as activity-guided fractionation, LC-MS/MS-based metabolite profiling, and NMR-guided compound isolation are instrumental in such investigations. Such investigations may inform the rational design of NLRP3-targeting agents and validate the feasibility of developing natural product-based molecular scaffolds for inflammasome inhibition. Furthermore, in vivo studies, such as those in zebrafish and murine models of NLRP3-driven diseases (e.g., MSU-induced peritonitis or high-fat diet–induced metabolic inflammation), are critical for determining the pharmacokinetic properties, safety profiles, and therapeutic efficacy of POE and its active derivatives. In parallel, evaluating the potential off-target effects of POE is essential to ensure its selectivity and avoid unintended immunological suppression. In summary, these findings indicate that POE suppresses NLRP3 inflammasome activation.

## Supplementary Information

Below is the link to the electronic supplementary material.

Supplementary Material 1

Supplementary Material 2

Supplementary Material 3

Supplementary Material 4

## Data Availability

The datasets generated and analyzed during this study are available from the corresponding author upon reasonable request.
