# Supplementary material for: Premna odorata extract exhibits anti-inflammatory activity via suppression of NLRP3 inflammasome
Source: Sci Rep. 2026 Jun 10;16:26467. doi: 10.1038/s41598-026-55217-1 (PMC13500559; doi:10.1038/s41598-026-55217-1)

Figure 1D Raw Data

pro-IL-1β

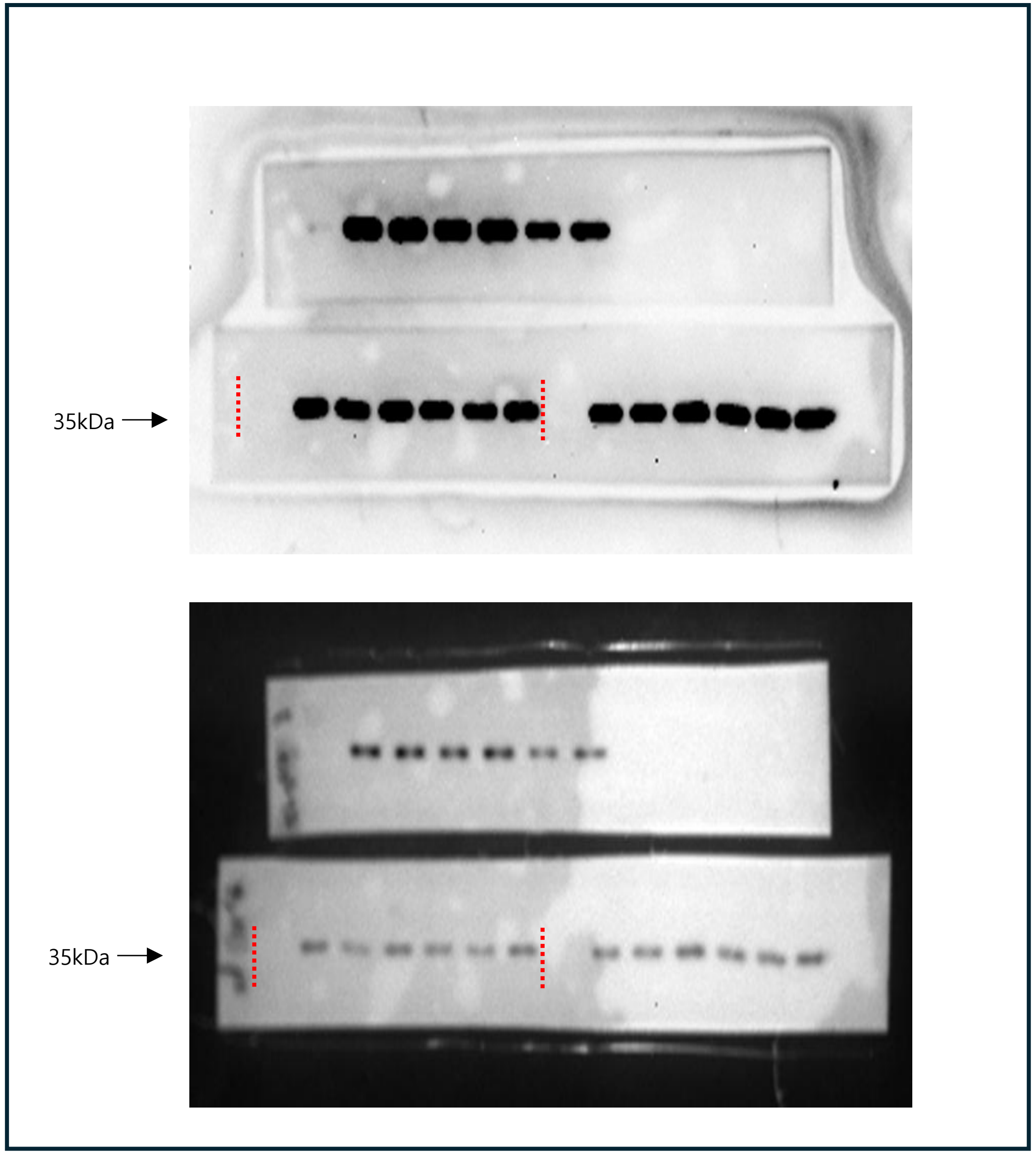

α-tubulin

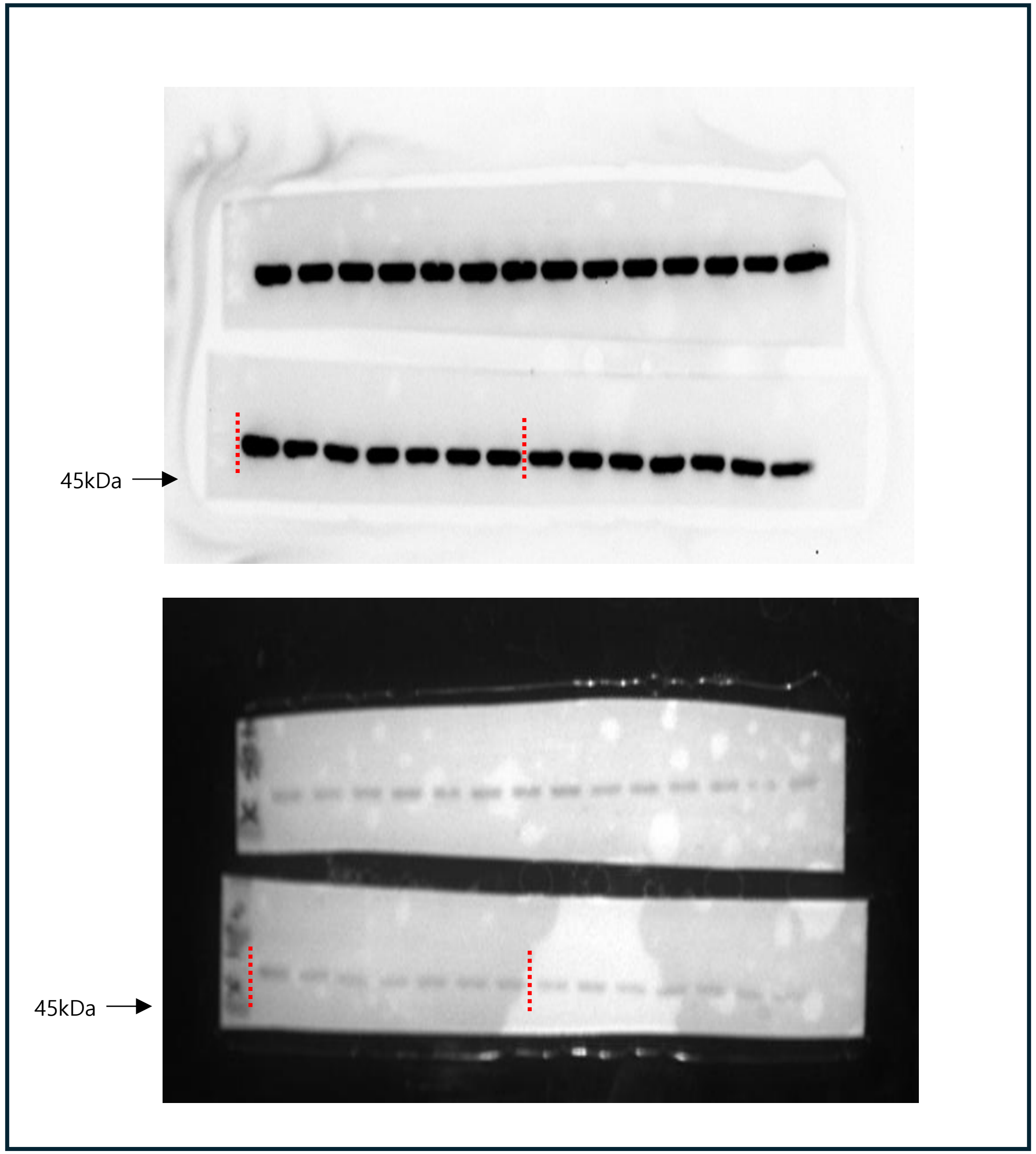

Figure 1F Raw Data

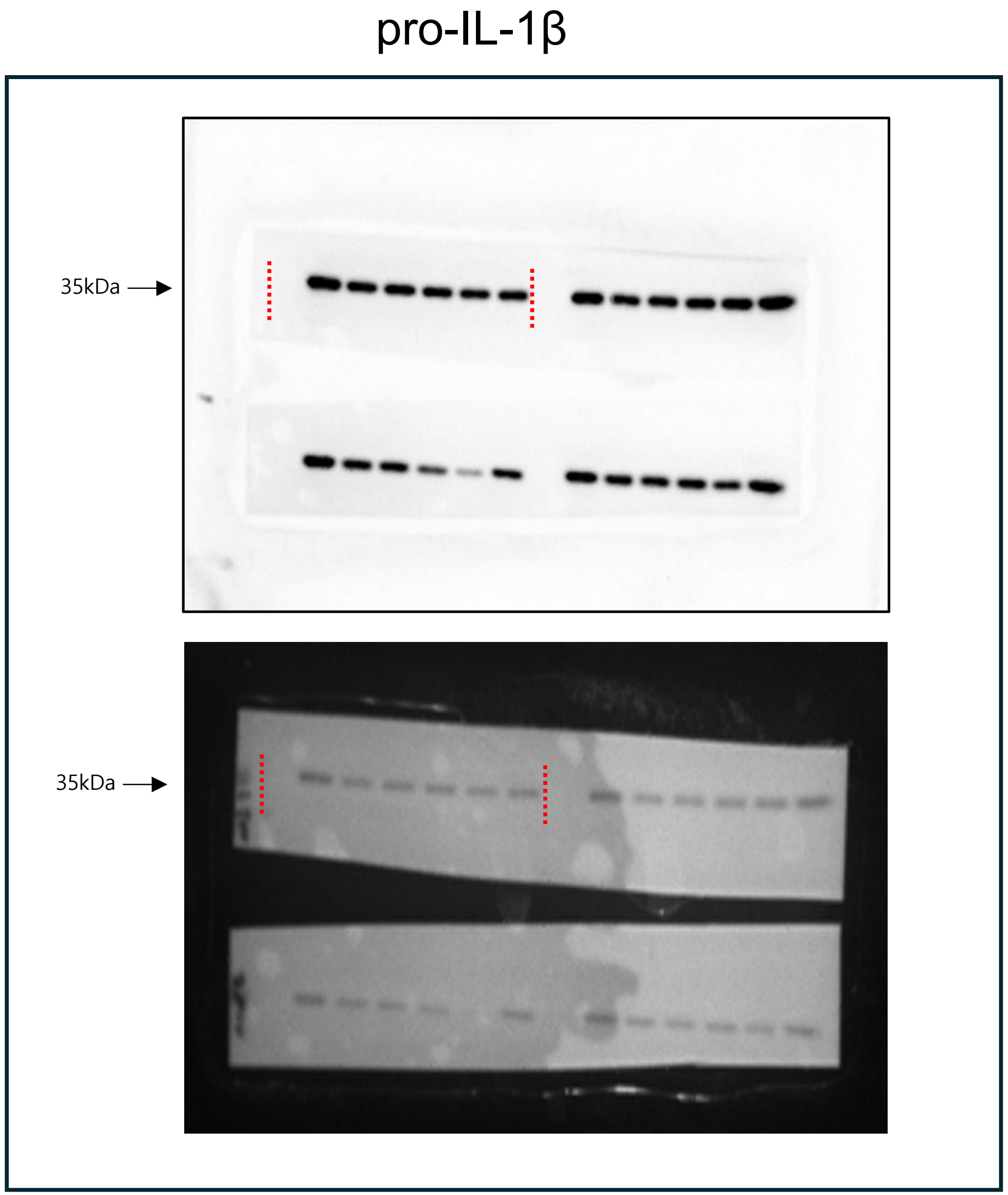

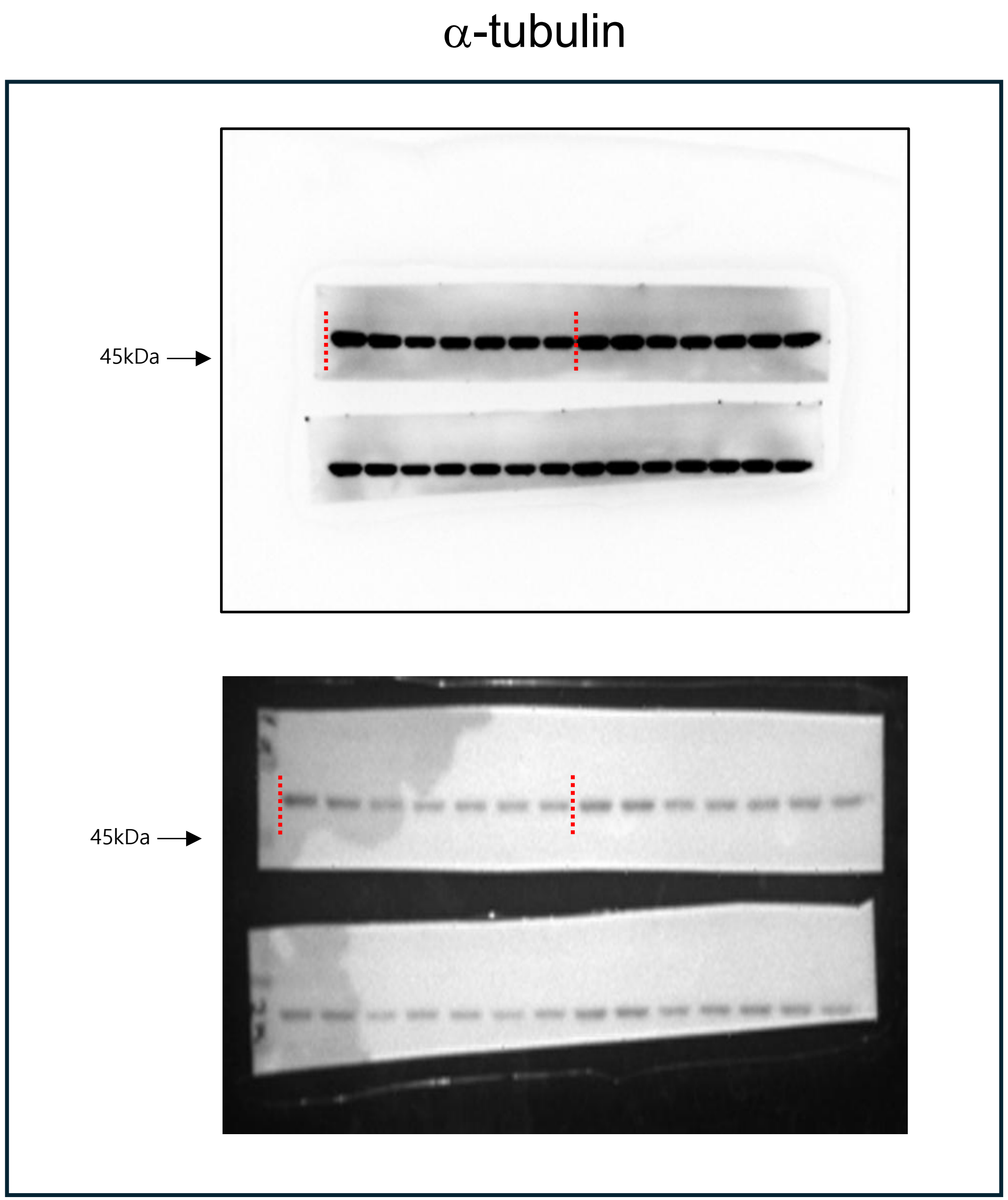

Figure 2A Raw Data

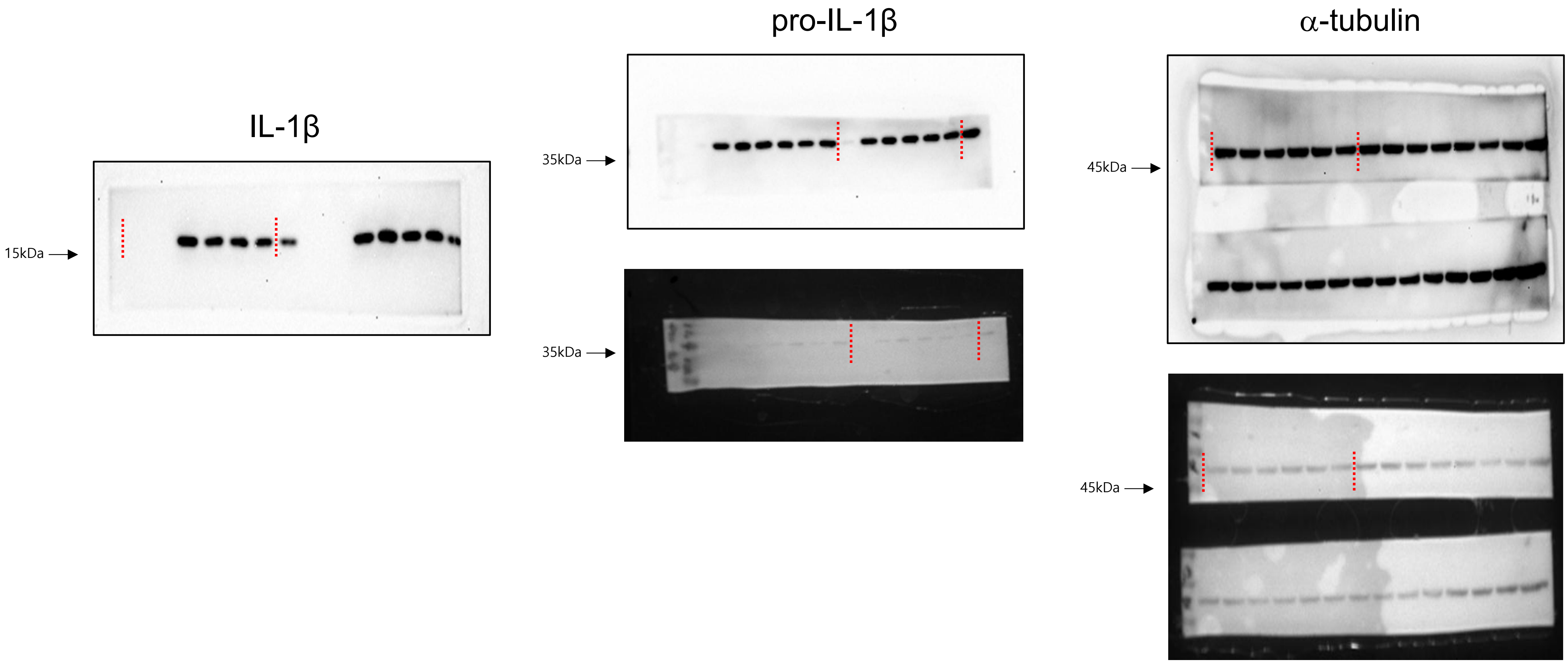

Figure 2B Raw Data

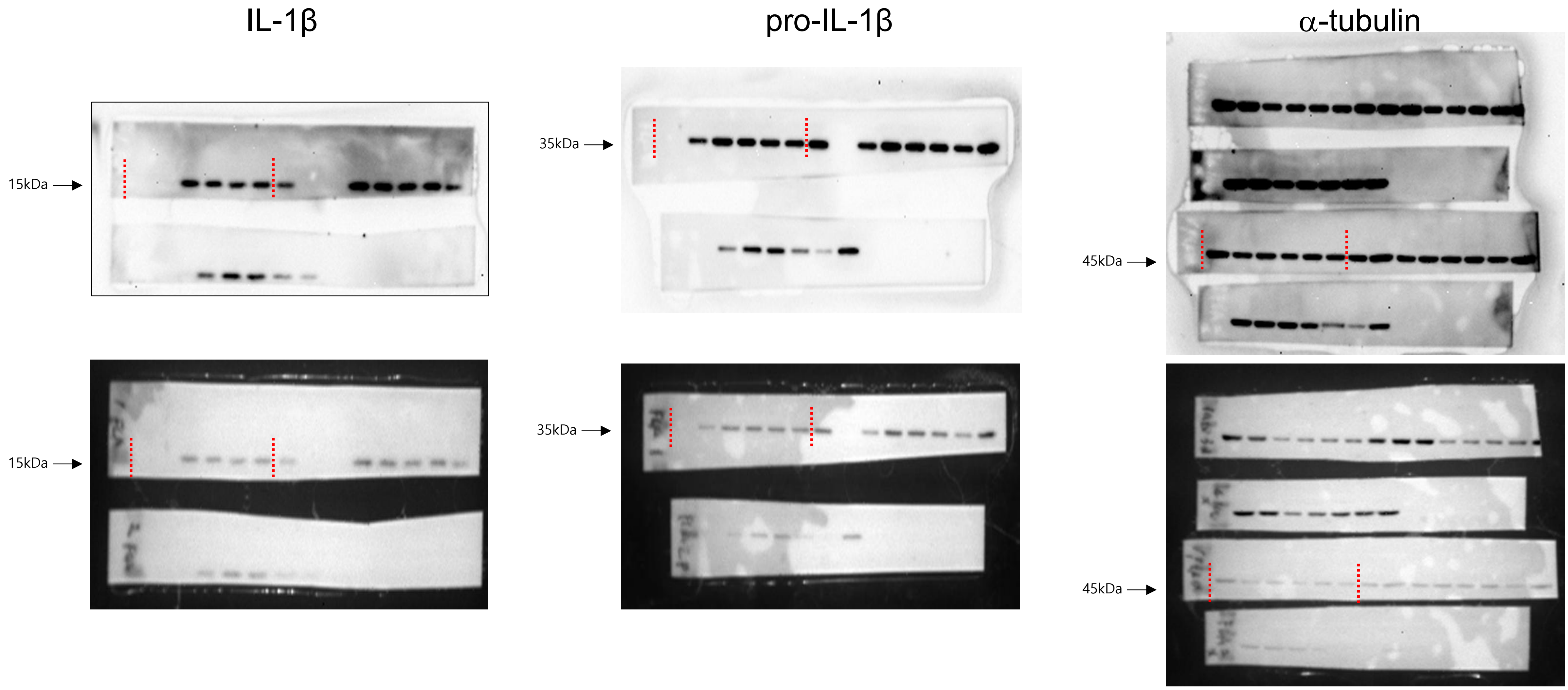

Figure 2C Raw Data

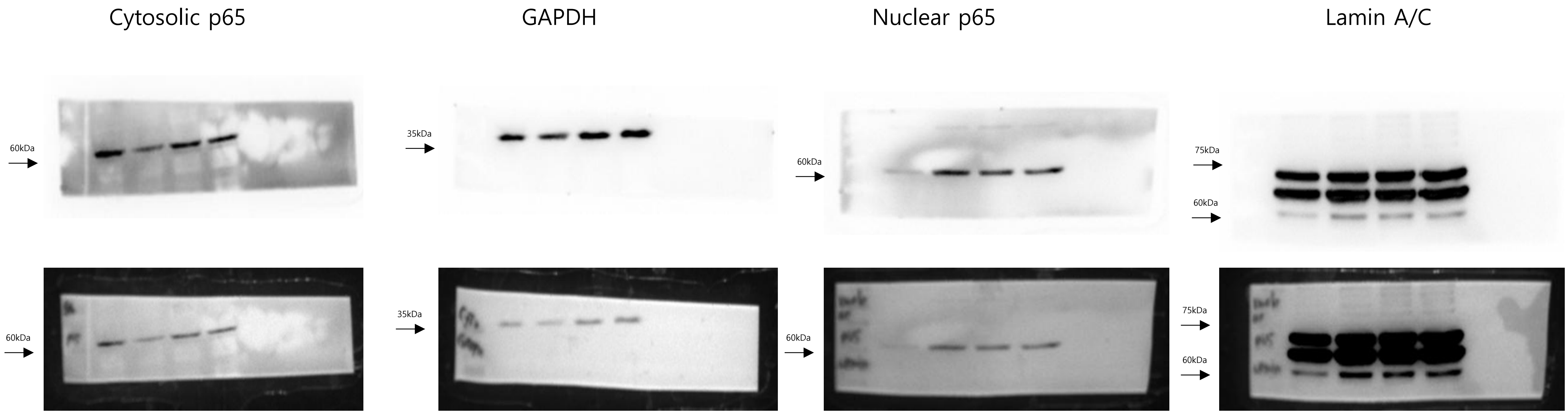

Figure 3A Raw Data

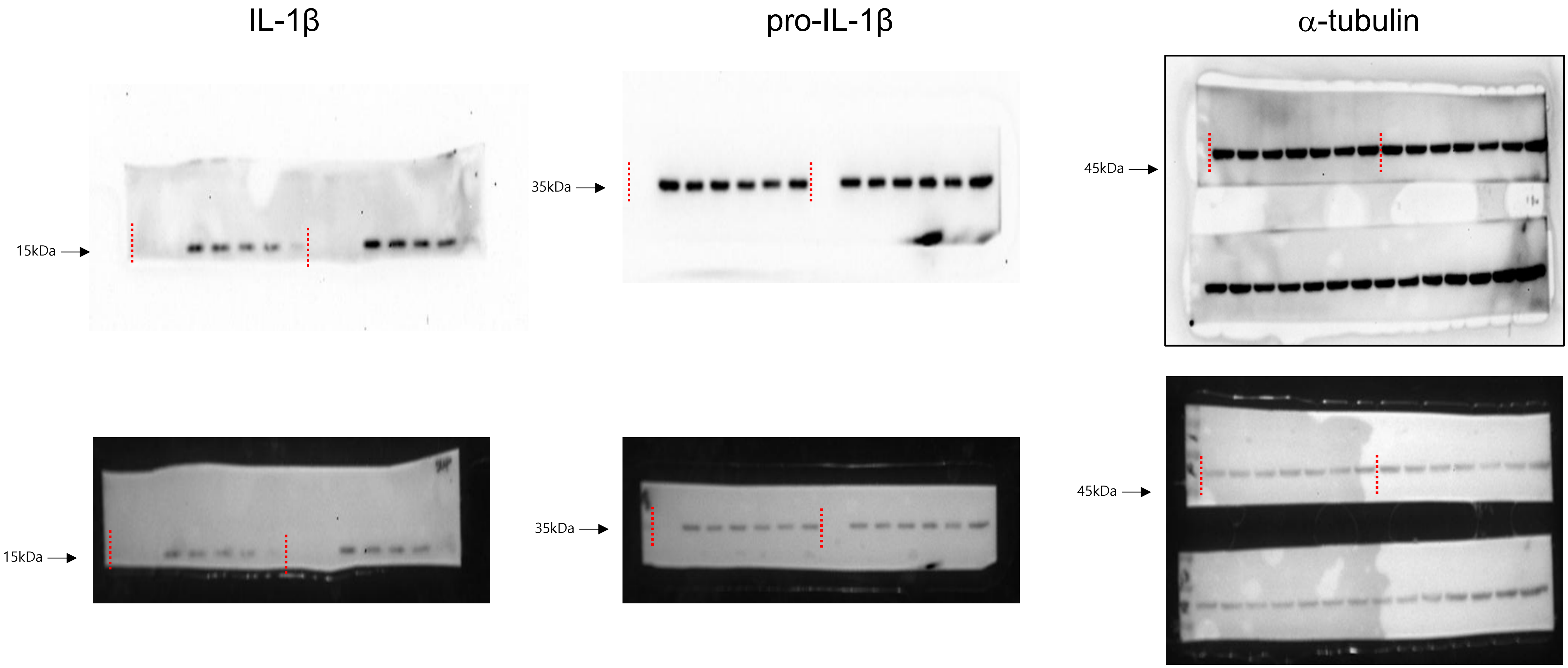

Figure 3D Raw Data

Lys caspase-1

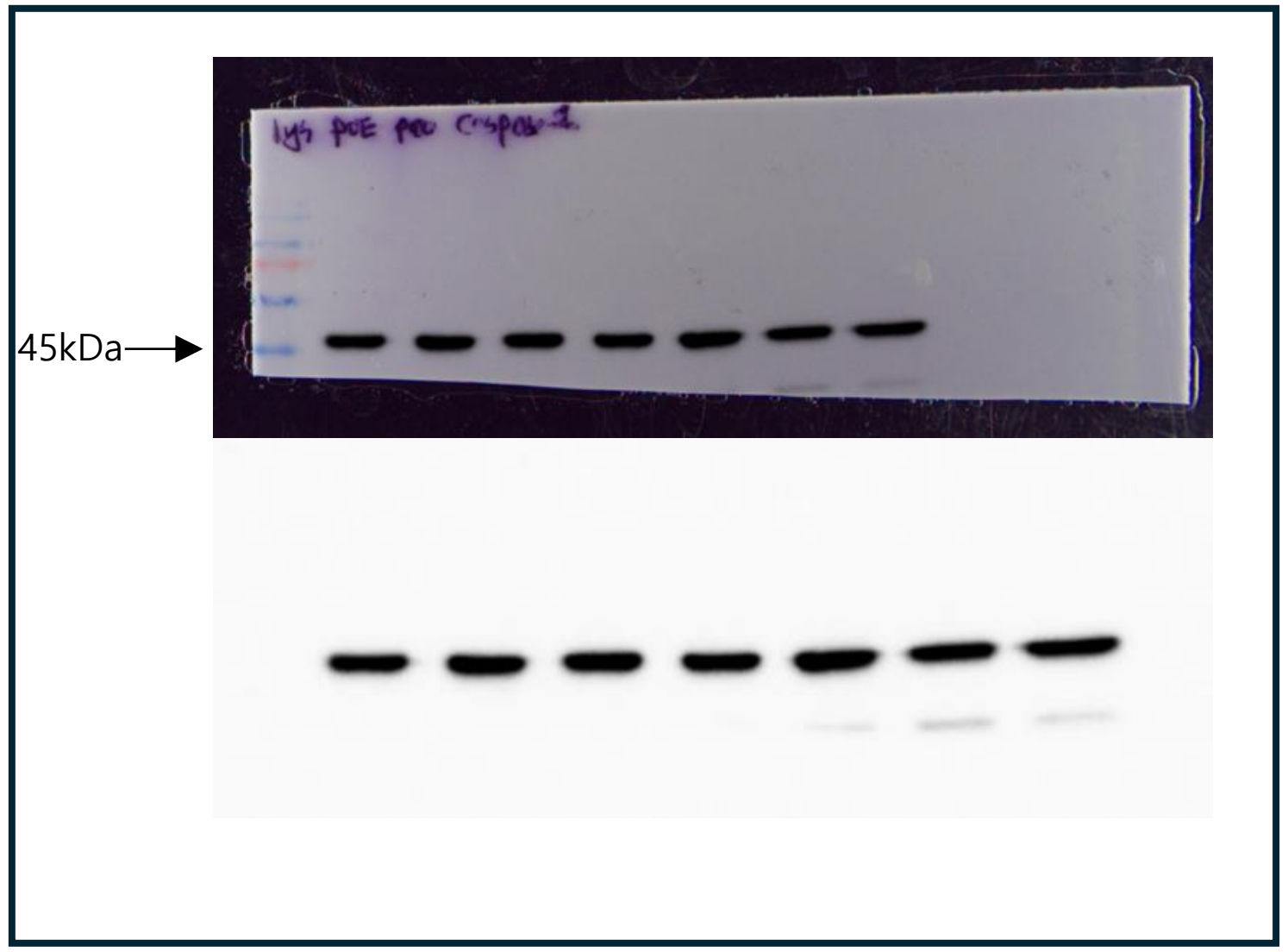

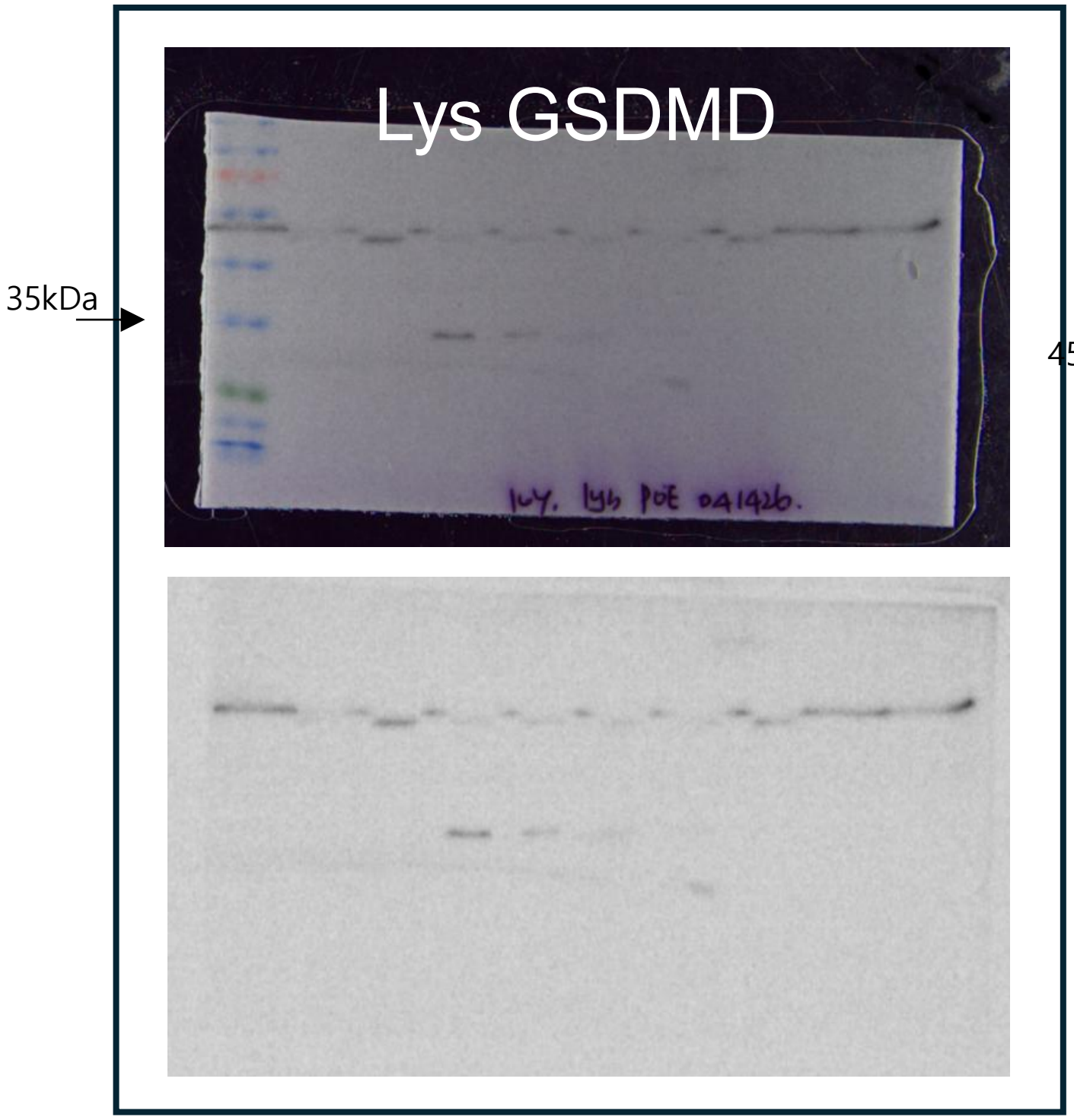

Lys α-tubulin

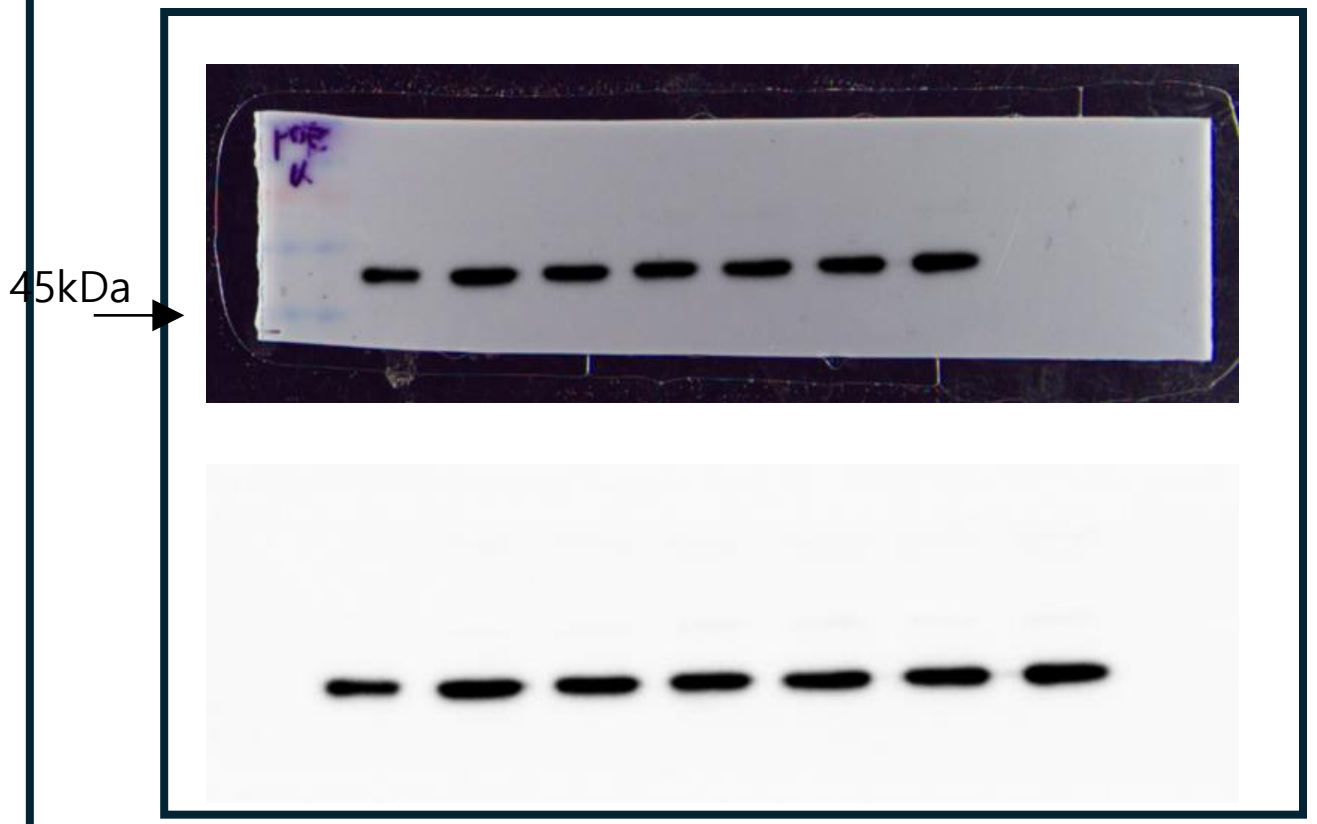

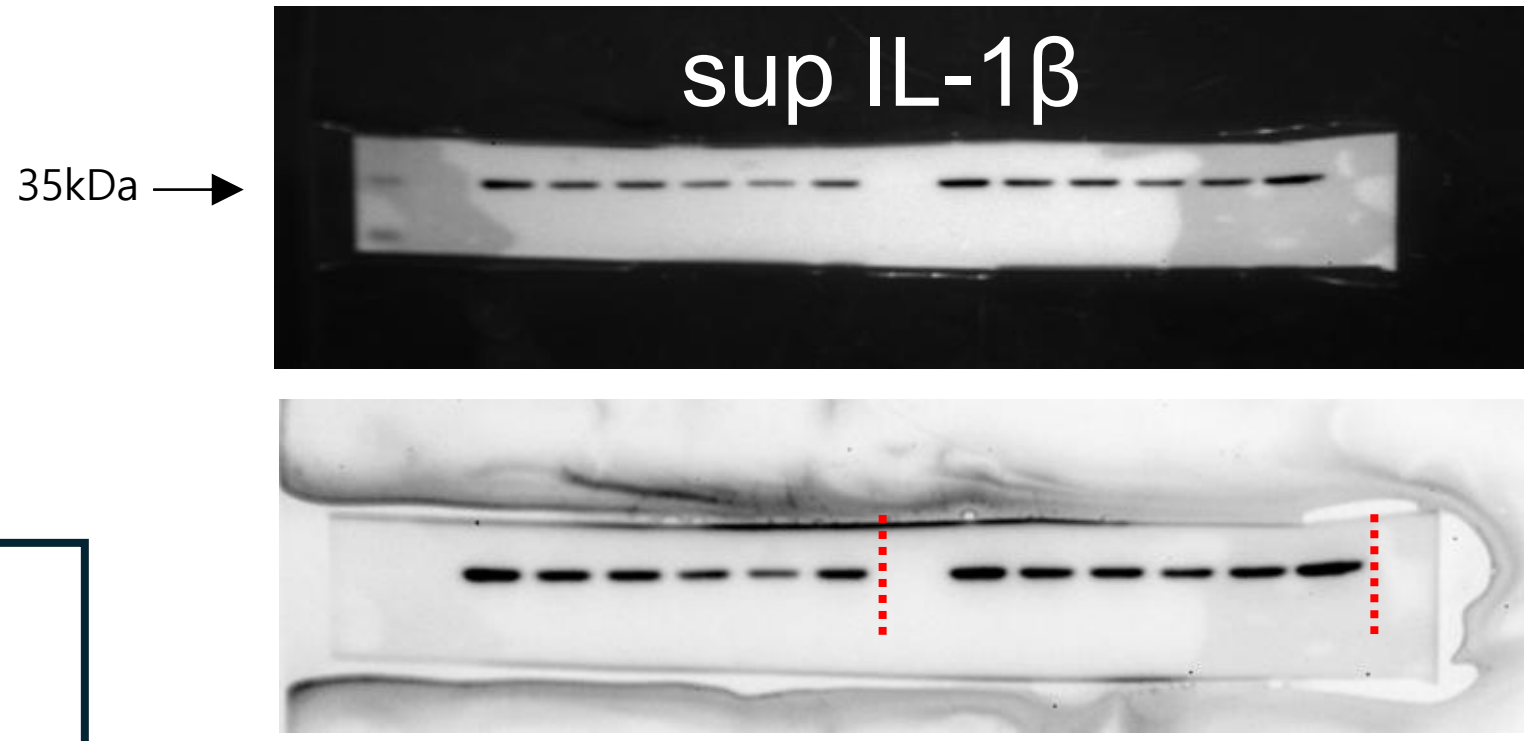

Lys IL-1β

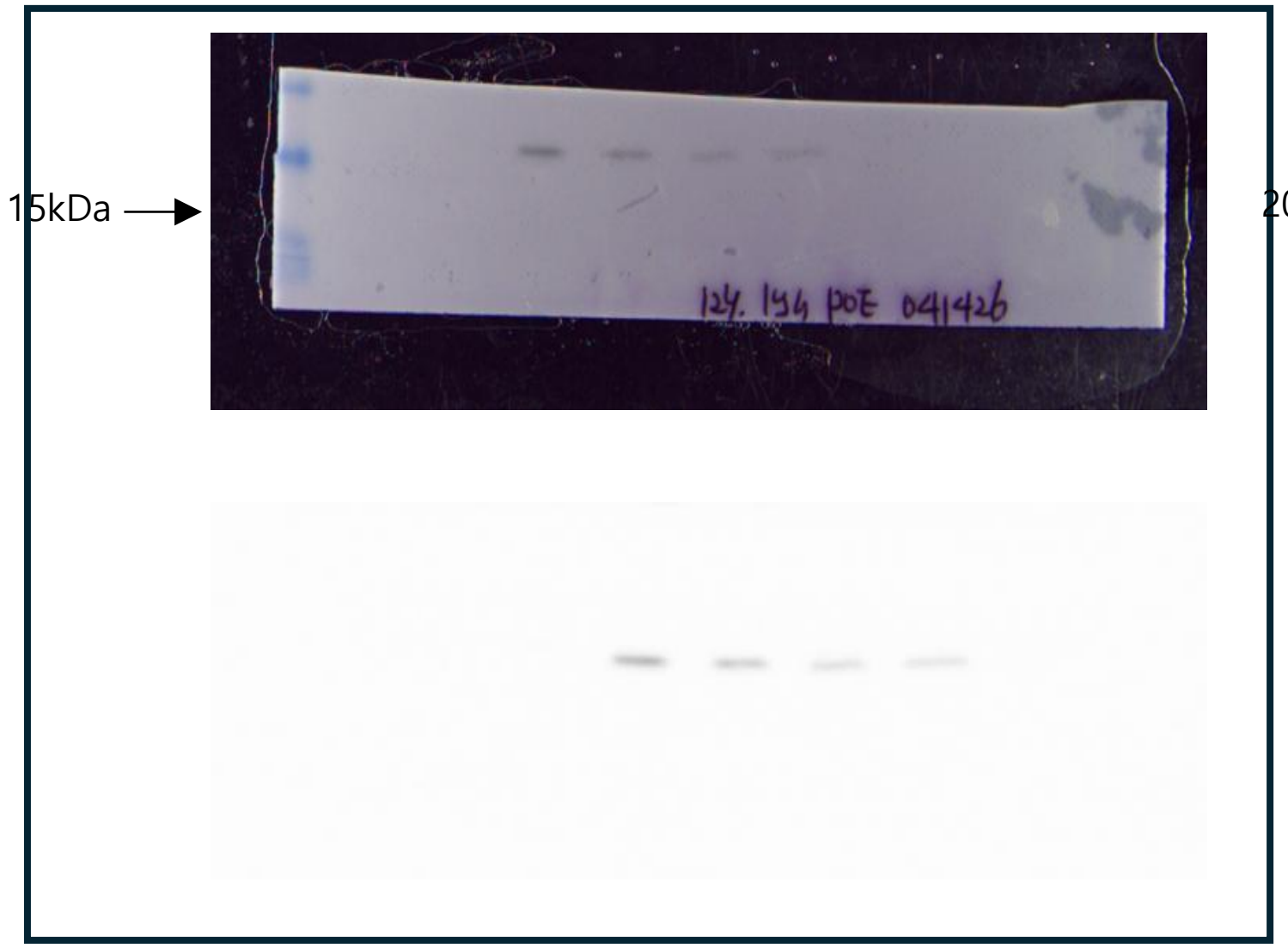

Sup caspase-1(p20)

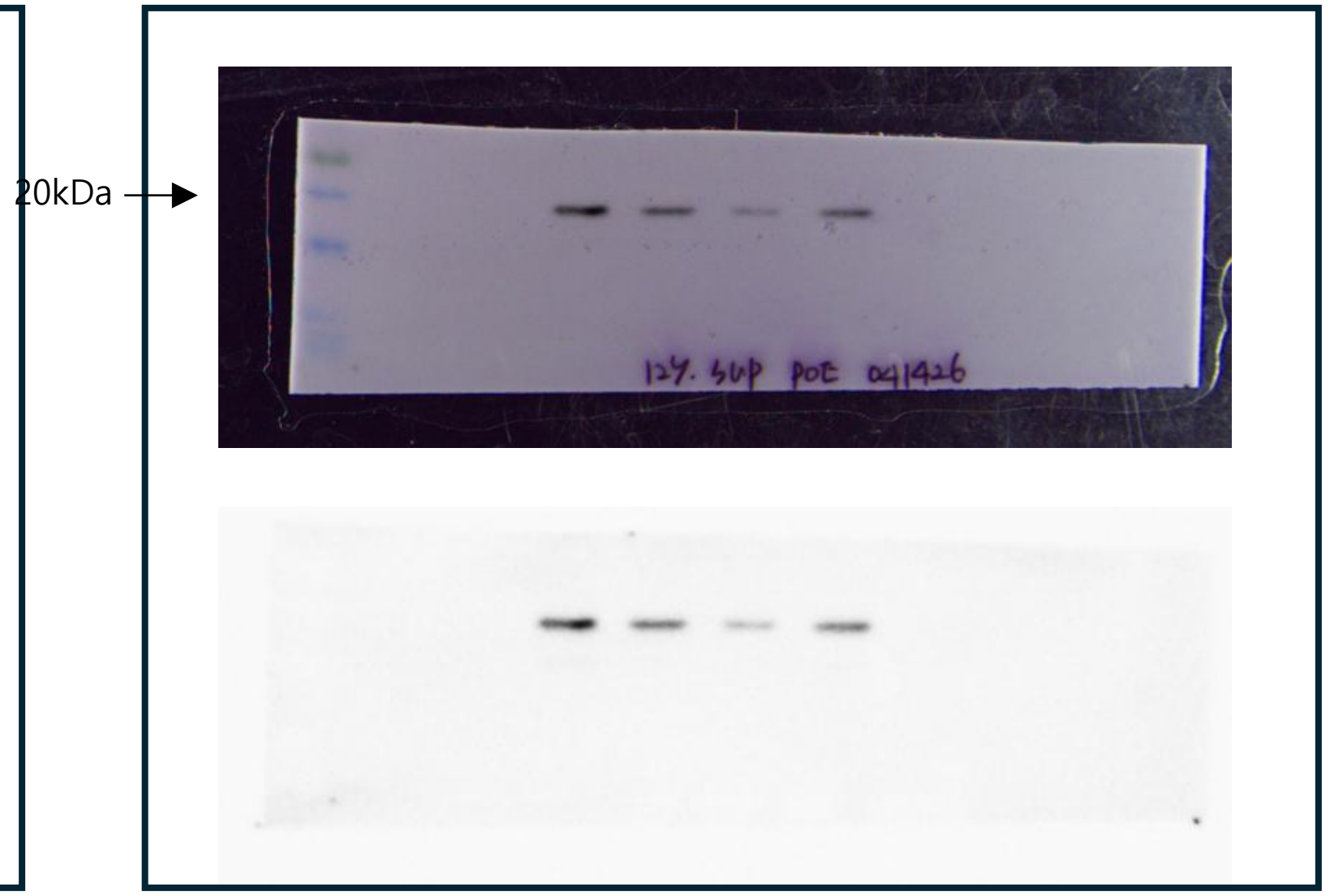

sup IL-1β

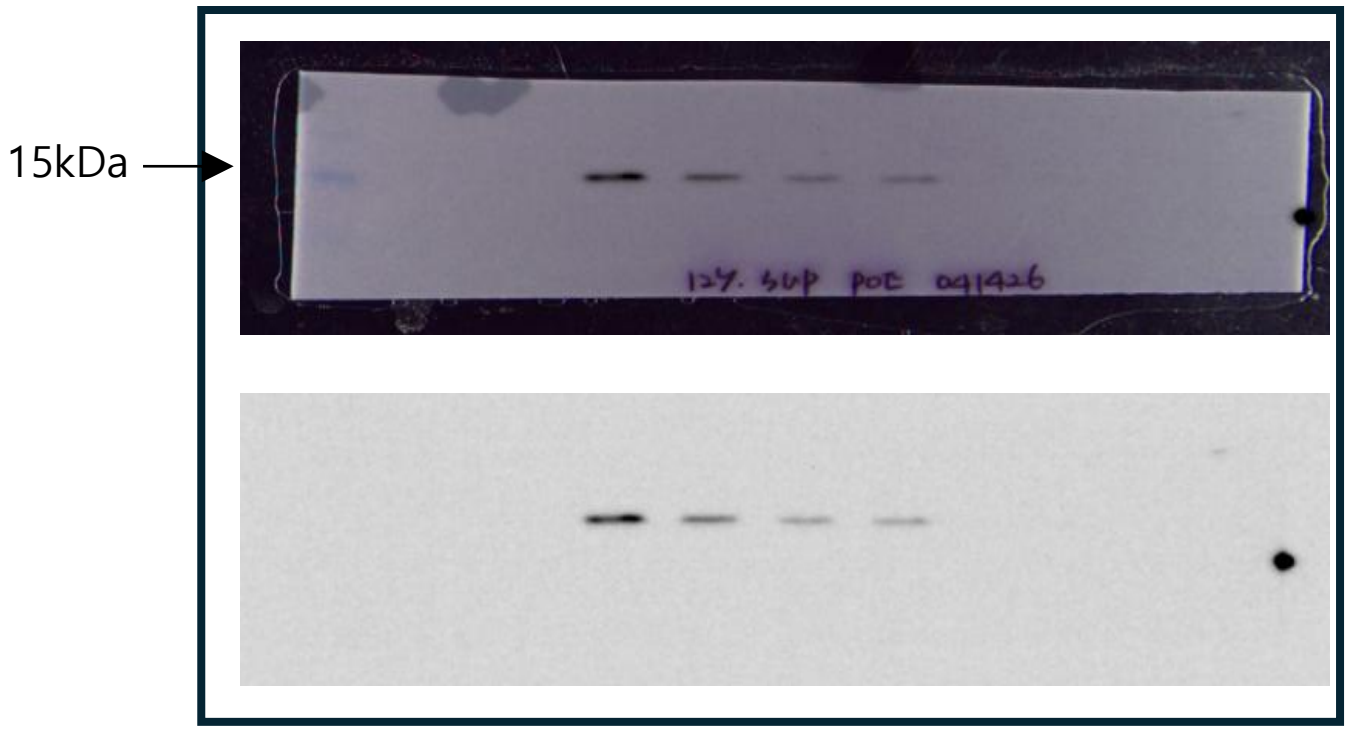

Figure 3E Raw Data

Lys α-tubulin

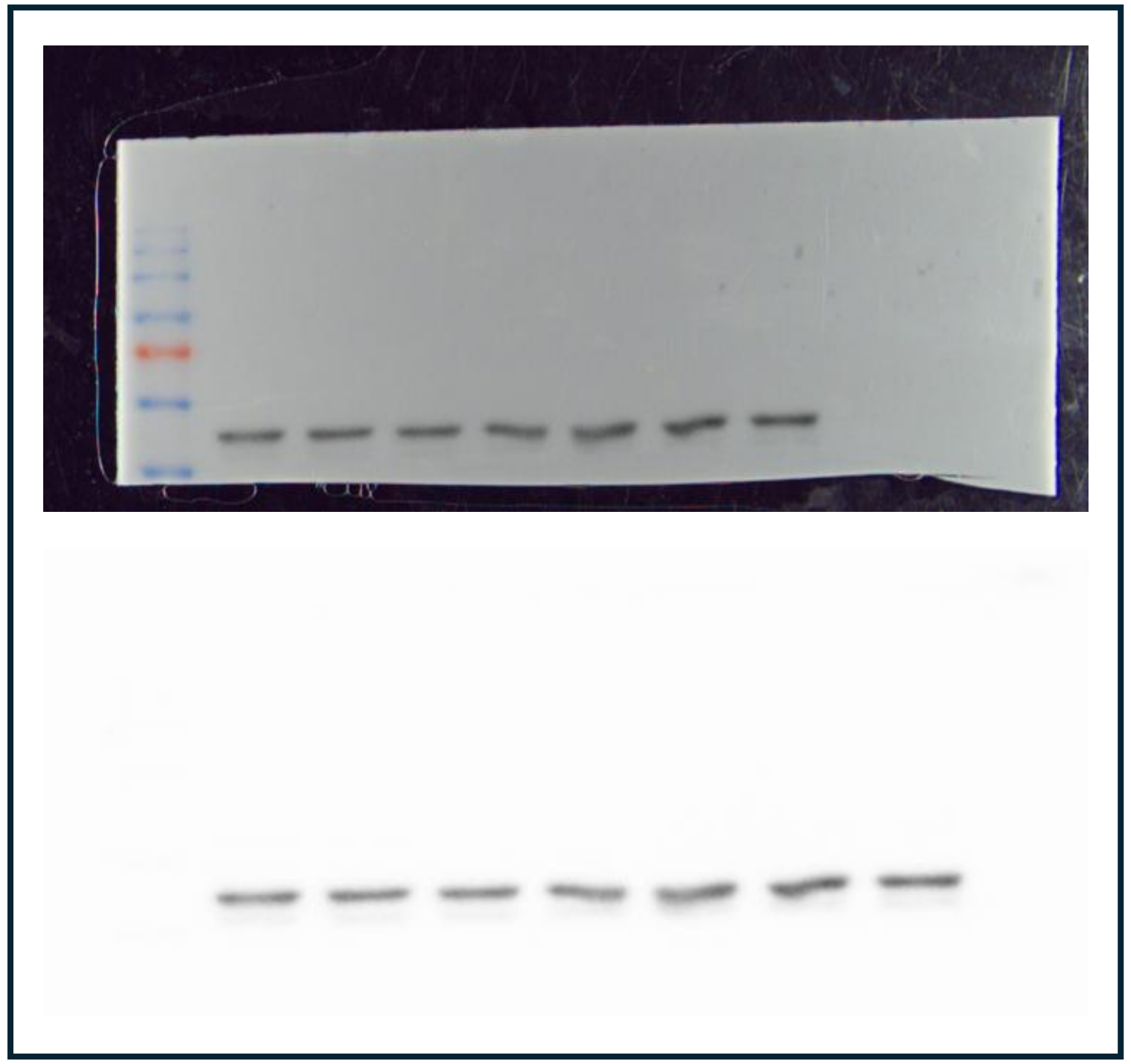

Lys pro-IL-1β

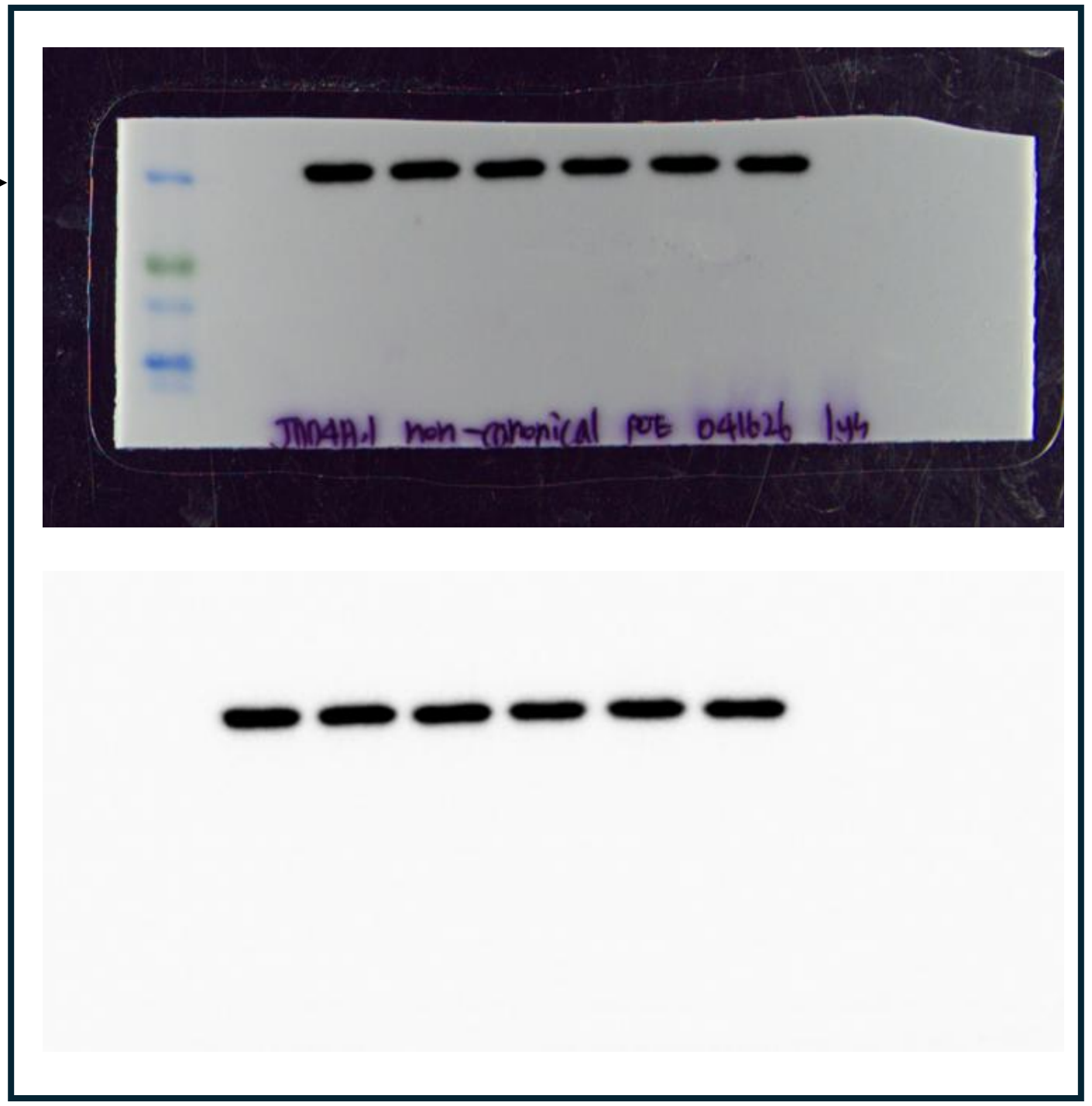

Sup IL-1β

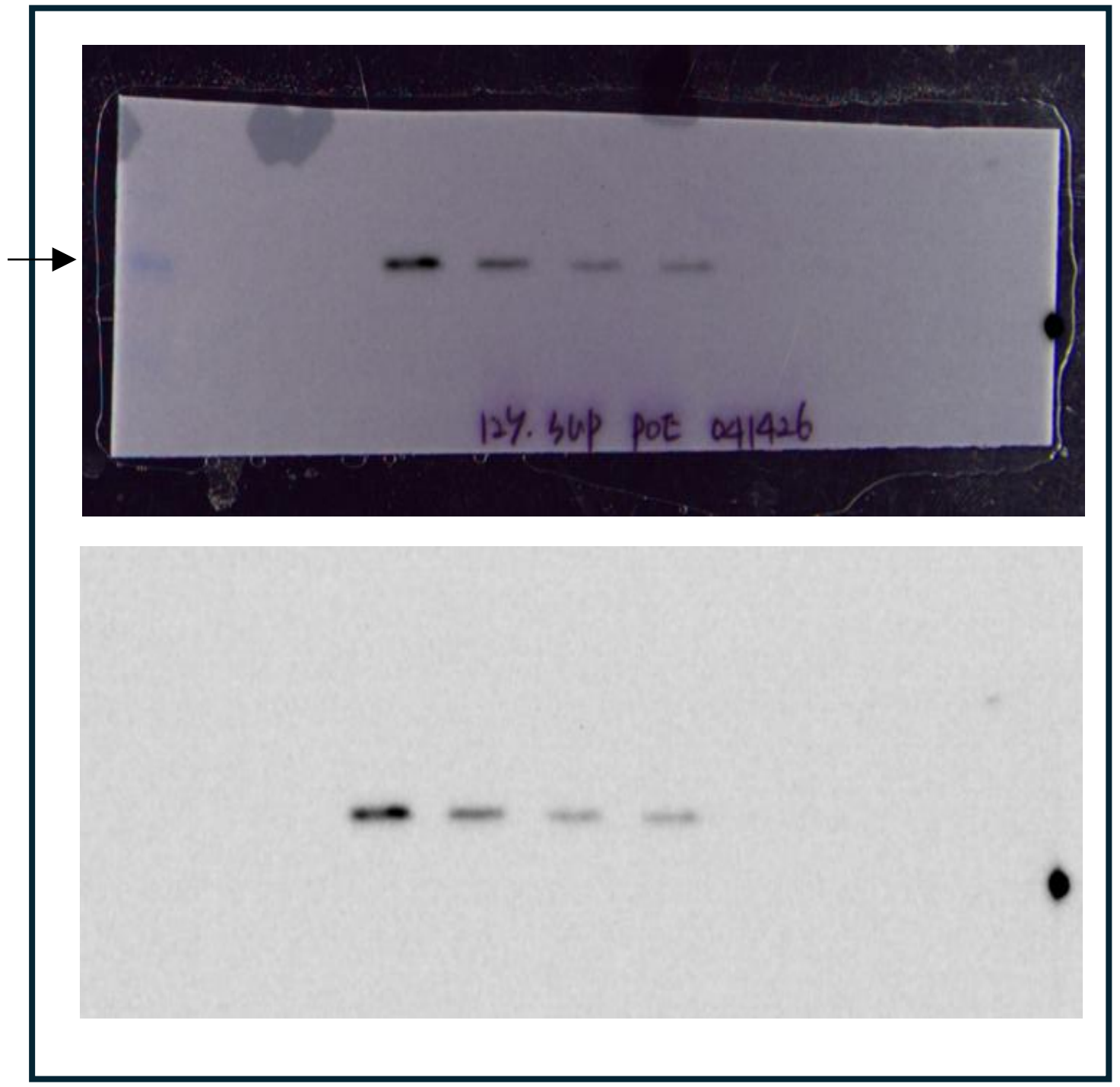

Figure 4C Raw Data

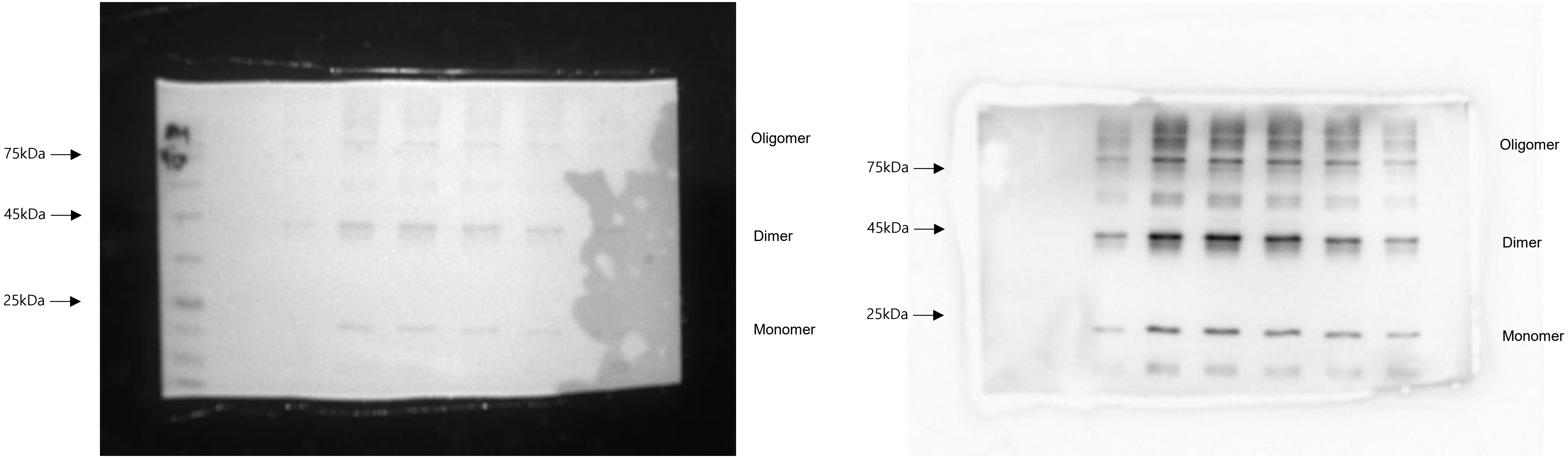

Supplement Figure 3 Raw Data

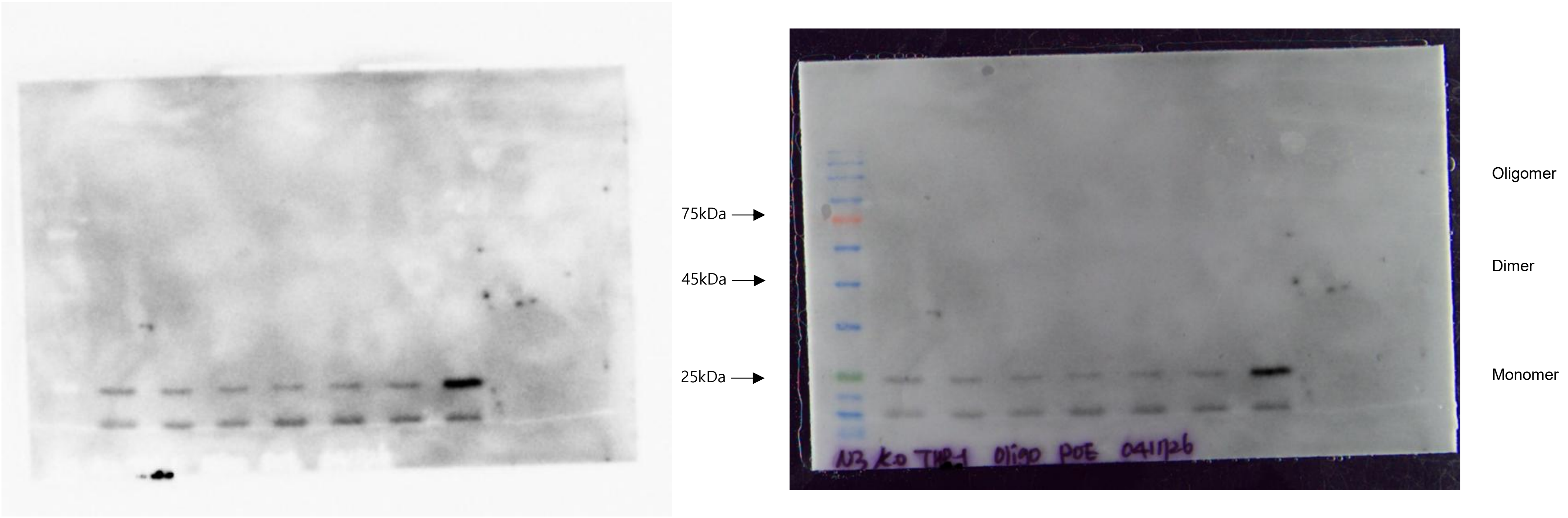

Supplement Figure 6 Raw Data

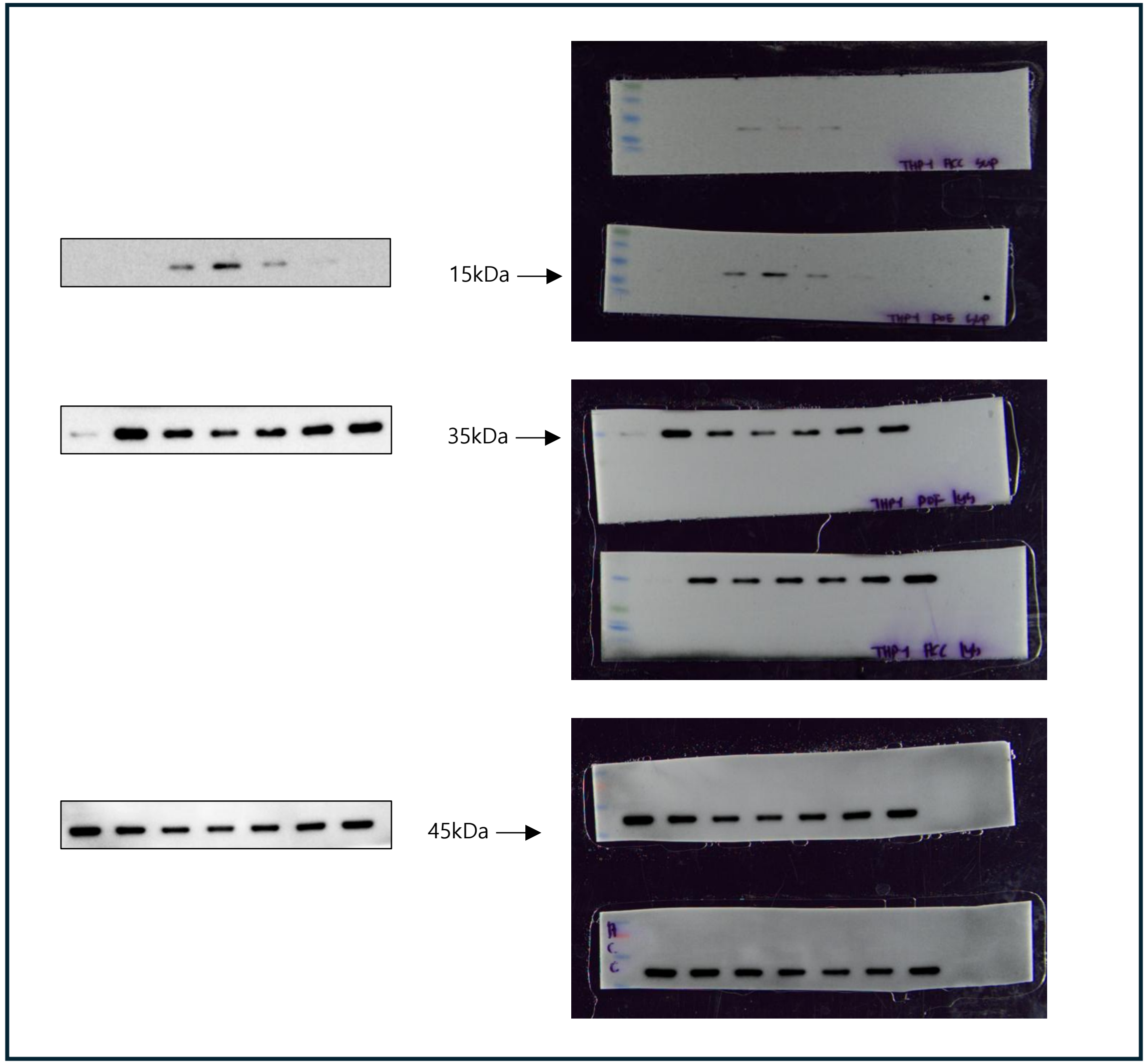

Supplement: Supplementary file 1 [file 41598_2026_55217_MOESM3_ESM.pdf]
