# Supplementary material for: Premna odorata extract exhibits anti-inflammatory activity via suppression of NLRP3 inflammasome
Source: Sci Rep. 2026 Jun 10;16:26467. doi: 10.1038/s41598-026-55217-1 (PMC13500559; doi:10.1038/s41598-026-55217-1)
Supplement: Supplementary file 2 [file 41598_2026_55217_MOESM4_ESM.pdf]

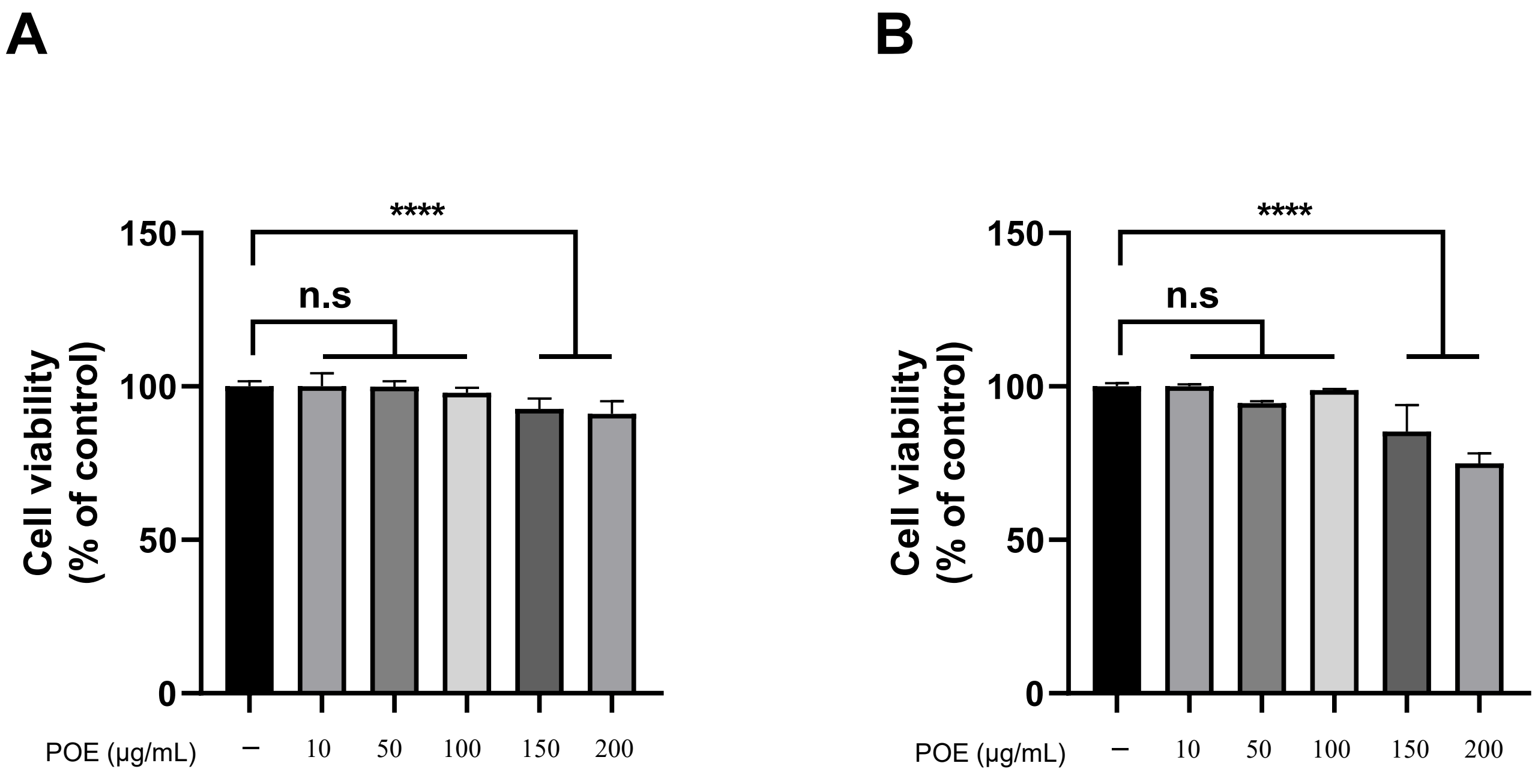

Supplementary Figure 1. Cytotoxicity of POE at higher concentrations in J774A.1 and THP-1 cells.

J774A.1 and THP-1 cells were treated with increasing concentrations of POE (10, 50, 100, 150, and 200 μg/mL) for 2 h or overnight, respectively, and cell viability was measured using the EZ-Cytox assay at 450 nm. Data are presented as mean ± SEM (n = 8). Statistical significance was determined using one-way ANOVA followed by Tukey's post hoc test. $^{*}p < 0.05$; $^{**}p < 0.01$; $^{***}p < 0.001$; $^{****}p < 0.0001$; n.s., not significant.

A

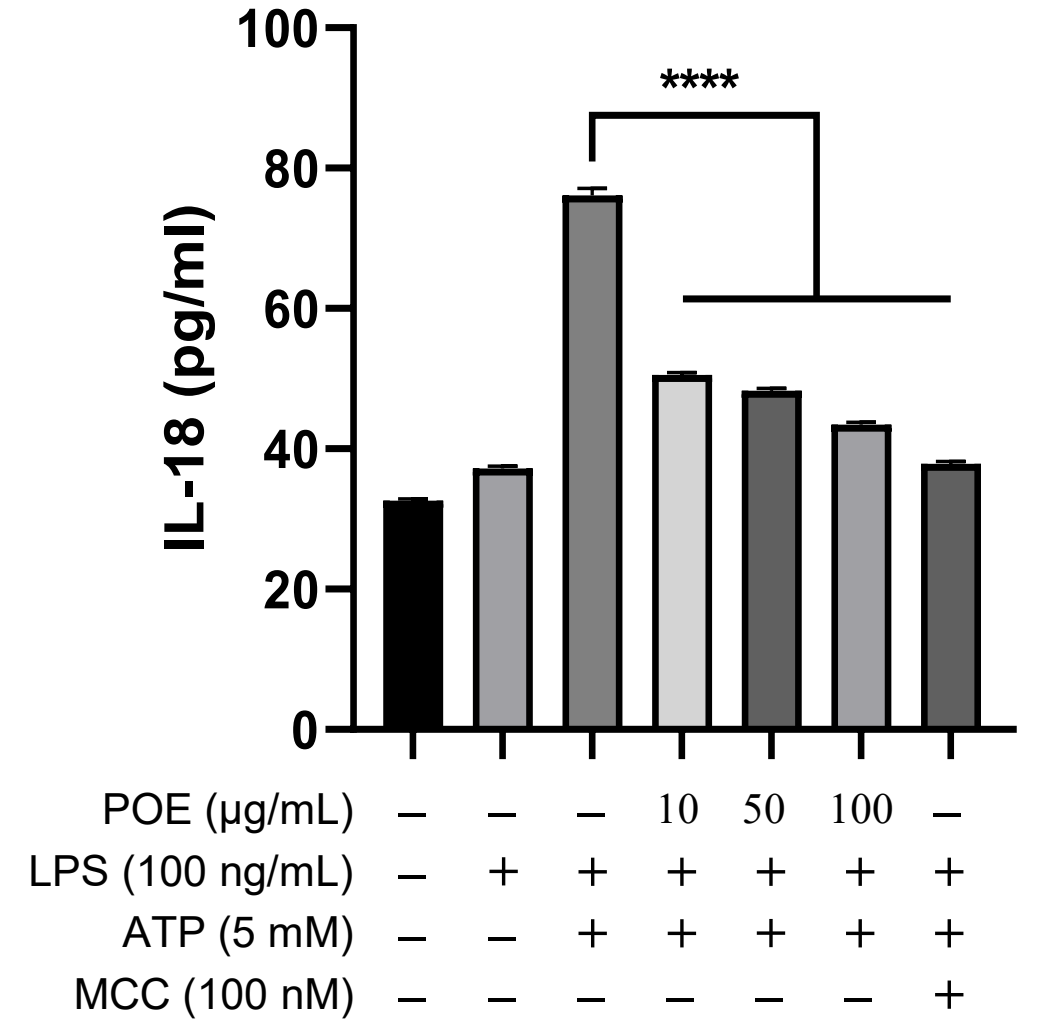

Supplementary Figure 2. POE suppresses IL-18 secretion in LPS-primed J774A.1 cells. LPS-primed J774A.1 cells were treated with concentrations of POE (10, 50, or 100 µg/mL) for 2 h and stimulated with ATP (5 mM) for 30 min. IL-18 levels in the supernatant were measured by ELISA. Data are presented as mean ± SEM (n = 3). Statistical significance was determined using one-way ANOVA followed by Tukey's post hoc test. *$p < 0.05$; **$p < 0.01$; ***$p < 0.001$; ****$p < 0.0001$; n.s., not significant.

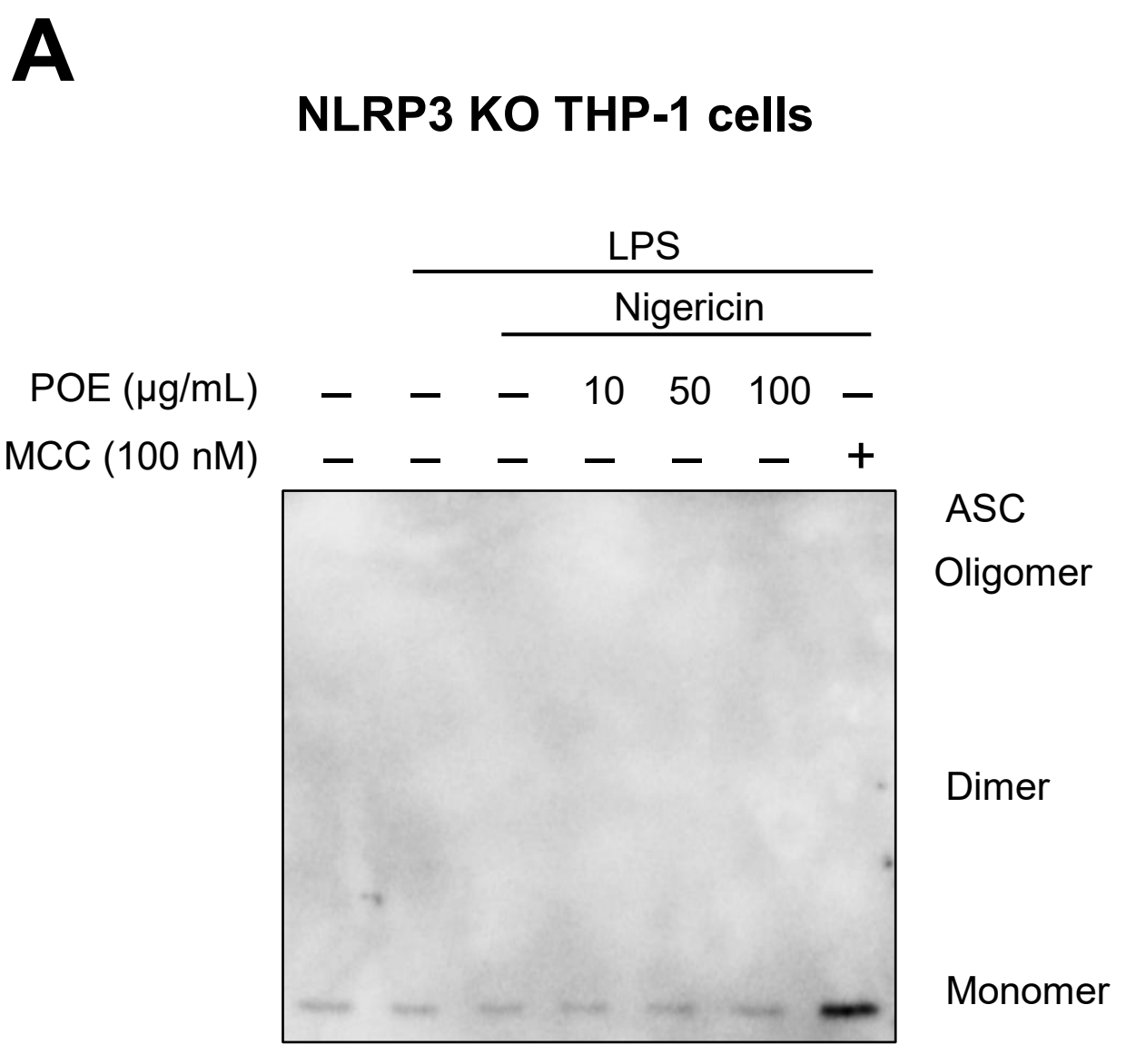

Supplementary Figure 3. ASC oligomerization is absent in NLRP3-knockout THP-1 cells regardless of POE treatment.

Differentiated NLRP3 Knockout (KO) THP-1 cell pellets were cross-linked using disuccinimidyl suberate (DSS, 25 mM), and ASC oligomerization was assessed by Western blot.

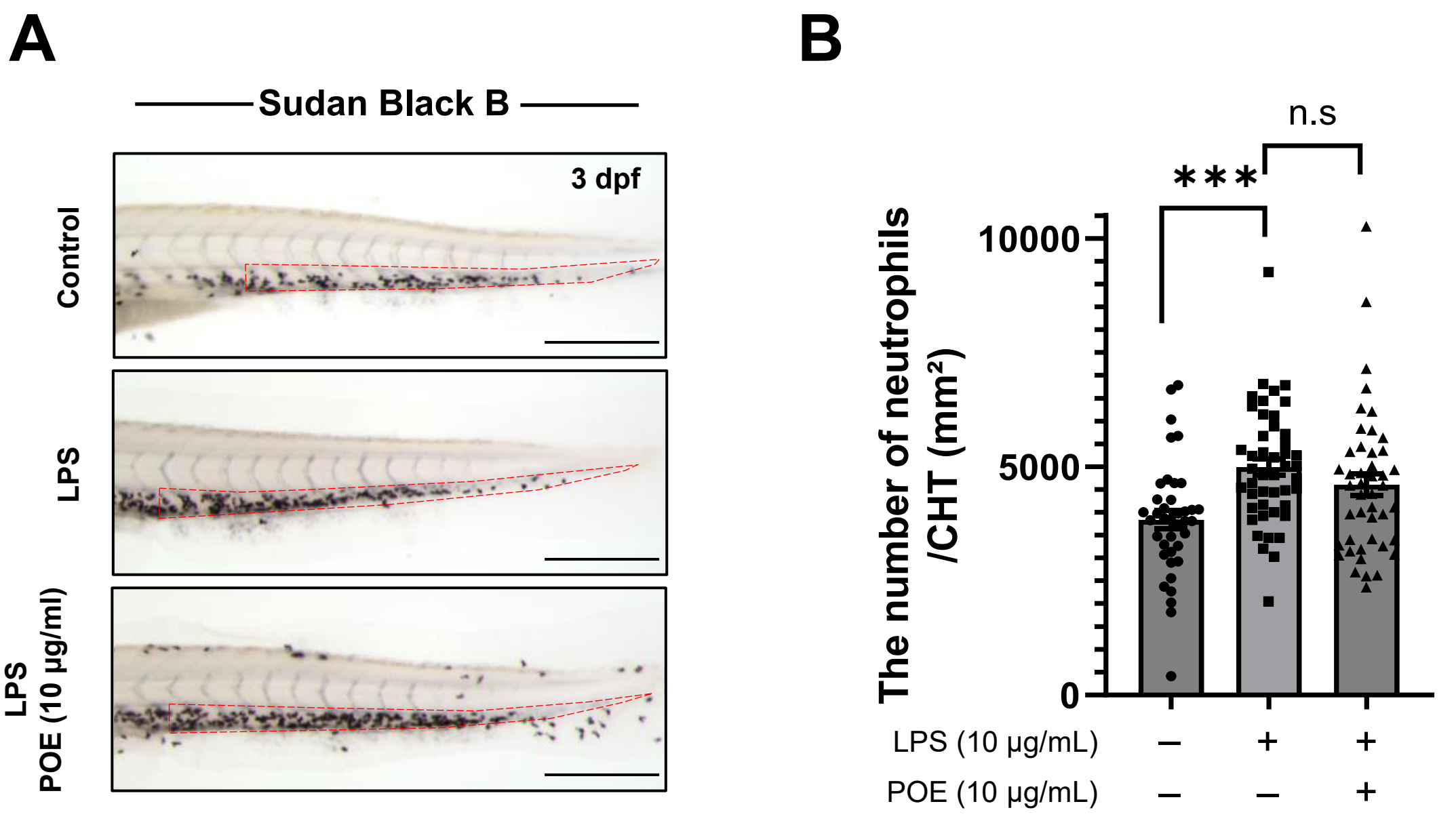

Supplementary Figure 4. POE demonstrates anti-inflammatory activity in zebrafish embryos. (A) Representative bright-field images of the CHT (red dashed line) in 3 dpf zebrafish embryos stained with Sudan Black B. (B) Quantification of neutrophil number within the CHT corresponding to (A), from two independent biological replicate experiments. The number of embryos: Control (n = 39), LPS (n = 46), and LPS + POE (n = 45). Control embryos were treated with 0.02 % DMSO in PTU, and LPS groups were treated with LPS (10 µg/mL) to induce inflammation, and LPS + POE groups were co-treated with LPS (10 µg/mL) and POE (10 µg/mL). Data are presented as mean ± SEM in each graph. The p values were calculated using one-way ANOVA followed by Tukey's post hoc HSD. ***$p < 0.001$; and n.s., not significantly different. Scale bar, 300µm.

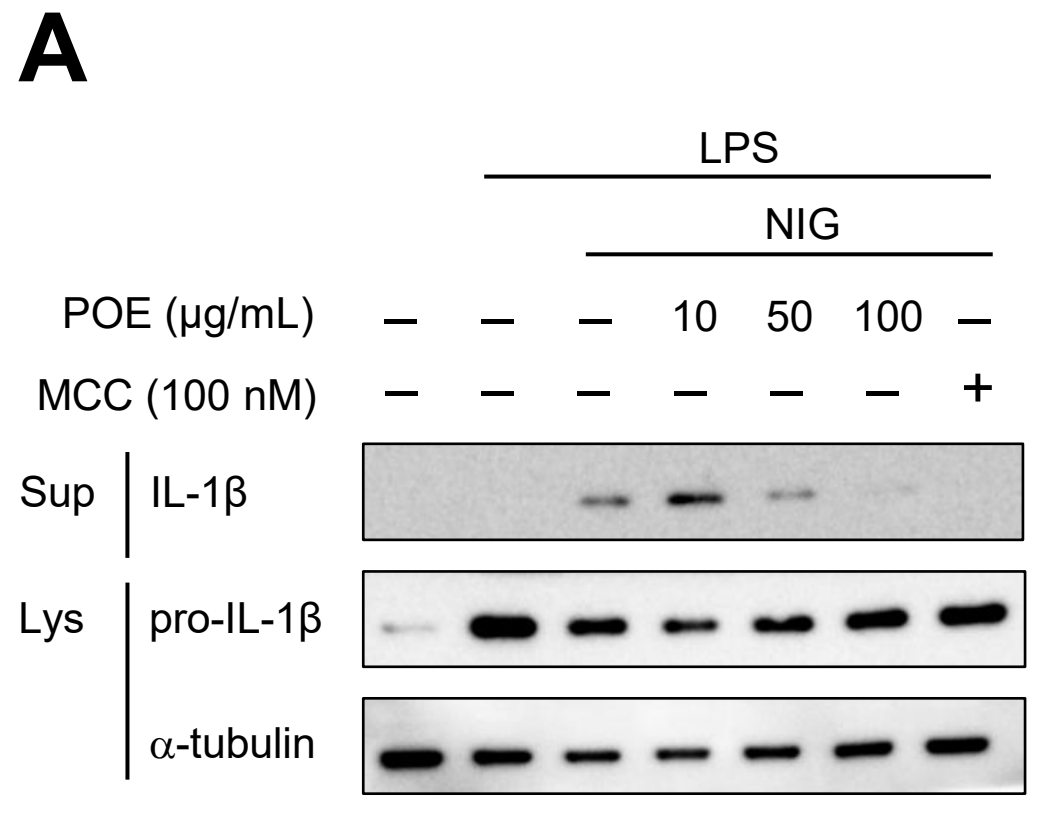

Supplementary Figure 5. **POE suppresses IL-1β secretion in LPS-primed THP-1 macrophages stimulated with nigericin.**

LPS (100 ng/mL)-primed differentiated THP-1 macrophages were treated with POE (10, 50, or 100 µg/mL) for 2 h and stimulated with nigericin (10 µM) for 30 min. IL-1β in the supernatant (Sup) and pro-IL-1β and α-tubulin in the cell lysate (Lys) were analyzed by Western blot. For each panel, all samples were run on the same gel and processed under identical experimental conditions.

Table S1. Tentative identification of compounds isolated from POE.

NO	RT (min)	Detected ion $[M-H]^-$	Calculated ion $[M-H]^-$	Error (ppm)	Fragments	Molecular formula	Tentative identification	Structure	Reference
1	0.62	191.0548	191.0556	-4.2	701, 539, 377, 191	$C_7H_{12}O_6$	quinic acid	Carboxylic acid derivated	Kim et al., 2007
2	0.81	203.0186	203.0192	-3.0	355, 290, 203	$C_7H_8O_7$	5-Oxo-1,3-dioxolane-4,4-diacetic acid	Carboxylic acid derivated	Takeuchi et al., 1999
3	0.96	217.0343	217.0348	-2.3	487, 217	$C_8H_{10}O_7$	Acetoxyacetic anhydride	Carboxylic acid derivated	Reva et al., 2004
4	2.07	387.0925 $[M+HCOO]^-$	387.0927 $[M+HCOO]^-$	-0.5	775, 387, 343	$C_{18}H_{16}O_7$	Pachypodol	Flavonol	Kim et al., 2019
5	2.79	243.0498	243.0505	-2.9	371, 243	$C_{10}H_{12}O_7$	Galloylglycerol	Phenolic	Zhang & Akoh, 2020
6	3.21	477.1609	477.1608	0.2	477, 431, 395, 353, 191	$C_{20}H_{30}O_{13}$	Kelampayoside A	Phenolic	Kitagawa et al., 1996
7	3.39	325.0913	325.0923	-3.1	651, 325, 163	$C_{15}H_{18}O_8$	Melilotoside	Phenolic	Afshar et al., 2017
8	3.70	387.1647	387.1655	-2.1	387, 289	$C_{18}H_{28}O_9$	Tuberonic acid glucoside	Monoterpene	Wakuta et al., 2010
9	3.85	593.1506	593.1506	0.0	593, 387	$C_{27}H_{30}O_{15}$	Vicenin 2	Flavone	Nguyen et al., 2025
10	4.10	563.1401	563.1401	0.0	563	$C_{26}H_{28}O_{14}$	Vicenin 3	Flavone	Nguyen et al., 2025
11	4.25	563.1398	563.1401	-0.5	1127, 563	$C_{26}H_{28}O_{14}$	Schaftoside	Flavone	Elmaidomy et al., 2019
12	4.35	563.1396	563.1401	-0.9	583, 563	$C_{26}H_{28}O_{14}$	Apigenin 6-*C*-*β*-xylopyranosyl-8-*C*-*β*-galactopyranoside	Flavone	Rayyan et al., 2010
13	4.42	563.1404	563.1401	0.5	563, 533	$C_{26}H_{28}O_{14}$	Vicenin 1	Flavone	Nguyen et al., 2025
14	4.56	533.1293	533.1295	-0.4	565, 533	$C_{25}H_{26}O_{13}$	Apigenin 6,8-di-*C*-*β*-D-arabinopyranoside	Flavone	Elmaidomy et al., 2019
15	4.70	533.1294	533.1295	-0.2	1067, 533	$C_{25}H_{26}O_{13}$	6-*C*-*α*-L-Arabinopyranosyl-8-*C*-*β*-D-xylopyranosylapigenin	Flavone	Rauter et al., 2007
16	4.87	533.1292	533.1295	-0.6	593, 565, 533	$C_{25}H_{26}O_{13}$	8-*C*-*α*-L-Arabinopyranosyl-6-*C*-*β*-D-xylopyranosylapigenin	Flavone	Nguyen et al., 2023
17	4.95	533.1297	533.1295	0.4	533	$C_{25}H_{26}O_{13}$	Apigenin 6,8-di-*C*-*α*-L-arabinopyranoside	Flavone	Elmaidomy et al., 2019
18	5.54	629.2663	629.2657	1.0	629, 565	$C_{26}H_{46}O_{17}$	Nonioside B	Sugar	Potterat et al., 2007
19	5.71	187.0966	187.0970	-2.1	563, 503, 187	$C_9H_{16}O_4$	Azelaic acid	Fatty acid	Ayorinde et al., 1988
20	7.83	327.2167	327.2171	-1.2	327, 235, 145, 116	$C_{18}H_{32}O_5$	(9*S*,10*E*,12*S*,13*S*,15*Z*)-9,12,13-Trihydroxy-10,15-octadecadienoic acid	Fatty acid	Hou, 1996
21	8.35	329.2321	329.2328	-2.1	329, 235, 116	$C_{18}H_{34}O_5$	Pinellic acid	Fatty acid	Gaurav et al., 2024
22	8.62	287.2215	287.2222	-2.4	327, 287, 235, 145, 116	$C_{16}H_{32}O_4$	10,16-Dihydroxyhexadecanoic acid	Fatty acid	Bakhsh et al., 2024
23	8.94	309.2068	309.2066	0.6	309, 116	$C_{18}H_{30}O_4$	Decanediol dimethacrylate	Fatty acid	Liu et al., 2012
24	9.21	359.0763	359.0767	-1.1	359, 309	$C_{18}H_{16}O_8$	Rosmarinic acid	Phenolic	Nguyen et al., 2025
25	9.40	307.1905	307.1909	-1.3	307, 116	$C_{18}H_{28}O_4$	Dihydrocapsiate	Phenolic	Nguyen et al., 2025
26	9.85	311.1853	311.1858	-1.6	311, 283, 116	$C_{17}H_{28}O_5$	1-(4-Methyl-7-oxooctyl) 1,2-cyclohexanedicarboxylate	Monoterpene	El Jezi, 2019
27	10.07	305.1748	305.1753	-1.6	305, 116	$C_{18}H_{26}O_4$	Dipentyl phthalate	Phthalate	Nguyen et al., 2025

Table S1. continued.

NO	RT (min)	Detected ion $[M-H]^-$	Calculated ion $[M-H]^-$	Error (ppm)	Fragments	Molecular formula	Tentative identification	Structure	Reference
28	12.15	293.2112	293.2117	-1.7	438, 393, 293, 235, 116	$C_{18}H_{30}O_3$	(9*Z*,11*E*,15*Z*)-13-Hydroxy-9,11,15-octadecatrienoic acid	Fatty acid	Dong et al., 2000
29	12.23	293.2115	293.2117	-0.7	438, 393, 293, 275, 116	$C_{18}H_{30}O_3$	(13*S*,9*Z*,11*E*,15*Z*)-13-Hydroxy-9,11,15-octadecatrienoic acid	Fatty acid	Dong et al., 2000
30	12.28	293.2111	293.2117	-2.0	438, 393, 293, 275, 116	$C_{18}H_{30}O_3$	17-Hydroxylinolenic acid	Fatty acid	Pohnert et al., 1999
31	12.33	293.2110	293.2117	-2.4	438, 393, 293, 235, 116	$C_{18}H_{30}O_3$	13-Oxo-9*Z*,11*E*-octadecadienoic acid	Fatty acid	Ko et al., 2023
32	12.42	293.2119	293.2117	0.7	438, 393, 293, 235, 116	$C_{18}H_{30}O_3$	9-Oxo-10*E*,12*Z*-octadecadienoic acid	Fatty acid	Zhao et al., 2015
33	12.67	291.1963	291.1960	1.0	309, 291, 265, 235, 116	$C_{18}H_{28}O_3$	Licanic acid	Fatty acid	Nguyen et al., 2025
34	13.18	295.2267	295.2273	-2.0	527, 395, 295, 116	$C_{18}H_{32}O_3$	13-Hydroxy-9,11-octadecadienoic acid	Fatty acid	Spiteller & Spiteller, 1997
35	13.27	295.2271	295.2273	-0.7	440, 395, 295, 116	$C_{18}H_{32}O_3$	(±)-Coriolic acid	Fatty acid	Nguyen et al., 2025
36	13.58	699.3810	699.3803	1.0	699, 473, 293, 116	$C_{32}H_{60}O_{16}$	Tetradecyl *O*-*β*-D-glucopyranosyl-(1→4)-*O*-*β*-D-glucopyranosyl-(1→4)-*β*-D-glucopyranoside	Glycosyl glyceride	Sharma et al., 2017
37	13.87	571.2885	571.2907	-3.9	571, 309, 116	$C_{32}H_{44}O_9$	Ganoderic acid H	Triterpene	Nguyen et al., 2025
38	14.18	297.2425	297.2430	-1.7	442, 397, 297, 116	$C_{18}H_{34}O_3$	Ricinoleic acid	Fatty acid	Altyar et al., 2020
39	14.45	481.2565	481.2590	-5.2	481, 295, 116	$C_{29}H_{38}O_6$	3-O-Methyl-22*β*,23-dihydroxy-6-oxotingenol	Triterpene	Neilson & Hynning, 1998
40	14.63	555.2834	555.2805	5.2	555, 295, 116	$C_{28}H_{44}O_{11}$	Picrasinoside F	Triterpene lactones	Elmaidomy et al., 2019
41	15.07	483.2728	483.2747	-3.9	505, 483, 421, 325, 116	$C_{29}H_{40}O_6$	1,2,3-Trideoxy-4,6:5,7-bis-*O*-[(4-propylphenyl)methylene]nonitol	Phenolic	Song et al., 2022
42	17.17	313.2739	313.2743	-1.3	473, 343, 313, 116	$C_{19}H_{38}O_3$	Methyl 12-hydroxystearate	Fatty acid	Wineburg & Swern, 1973
43	17.28	343.2843	343.2848	-1.5	473, 343, 116	$C_{20}H_{40}O_4$	Heptadecanoic acid, 2,3-dihydroxypropyl ester	Fatty acid	Ahmed et al., 2020
44	17.70	637.4313	637.4316	-0.5	637, 591, 473, 116	$C_{36}H_{62}O_9$	Mogroside I-E1	Triterpene	Liu et al., 2018
45	17.81	313.2742	313.2743	-0.3	473, 313, 116	$C_{19}H_{38}O_3$	Cetyl lactate	Fatty acid	Unno et al., 2020
46	17.87	410.3632	410.3634	-0.5	473, 410, 116	$C_{25}H_{49}NO_3$	5-[(2-Hydroxyethyl)amino]pentyl (9*Z*)-9-octadecenoate	Fatty acid	Inventor et al., 2024
47	18.14	327.2892	327.2899	-2.1	473, 395, 327, 299, 116	$C_{20}H_{40}O_3$	Glycol monostearate	Fatty acid	Martin & Lutton, 1965
48	18.26	355.1573	355.1545	7.9	610, 473, 355, 116	$C_{21}H_{24}O_5$	Kadsurenone	Benzofuran	Kim et al., 2011
49	18.44	653.4255	653.4265	-1.5	653, 381, 116	$C_{36}H_{62}O_{10}$	Cyclounifolioside C	Triterpene	Elmaidomy et al., 2019
50	19.68	383.1886	383.1858	7.3	383, 116	$C_{23}H_{28}O_5$	21-(Acetyloxy)-17-hydroxypregna-1,4,9(11)-triene-3,20-dione	Steroid	Cavina et al., 1992
