# Supplementary material for: Premna odorata extract exhibits anti-inflammatory activity via suppression of NLRP3 inflammasome
Source: Sci Rep. 2026 Jun 10;16:26467. doi: 10.1038/s41598-026-55217-1 (PMC13500559; doi:10.1038/s41598-026-55217-1)
Supplement: Supplementary file 3 [file 41598_2026_55217_MOESM2_ESM.docx]

**Supplementary results**

***Carboxylic acid derivatives***

Three low-molecular-weight carboxylic acid derivatives were identified from the methanolic extract of Premna odorata. Quinic acid (**1**; RT = 0.62 min; m/z 191.0548 [M–H]⁻), 5-oxo-1,3-dioxolane-4,4-diacetic acid (**2**; RT = 0.81 min; m/z 203.0186 [M–H]⁻), and acetoxyacetic anhydride (**3**; RT = 0.96 min; m/z 217.0343 [M–H]⁻) were tentatively assigned based on their accurate masses and retention times. These metabolites are commonly observed intermediates in plant primary organic-acid metabolism and serve as precursors for shikimate and phenolic biosynthesis.

***Flavonoids***

Nine flavonoid compounds were detected and classified as flavonols and flavones. Pachypodol (**4**; RT = 2.07 min; m/z 387.0925 [M+HCOO]⁻) was identified as a methoxylated flavonol. Among the C-glycosyl flavones, vicenin 2 (**9**; RT = 3.85 min; *m/z* 593.1506 [M–H]⁻), vicenin 3 (**10**; RT = 4.10 min; *m/z* 563.1401 [M–H]⁻), schaftoside (**11**; RT = 4.25 min; *m/z* 563.1398 [M–H]⁻), and apigenin 6-*C*-*β*-xylopyranosyl-8-*C*-*β*-galactopyranoside (**12**; RT = 4.35 min; *m/z* 563.1396 [M–H]⁻) were observed. Vicenin 1 (**13**; RT = 4.42 min; *m/z* 563.1404 [M–H]⁻), apigenin 6,8-di-*C*-*β*-D-arabinopyranoside (**14**; RT = 4.56 min; *m/z* 533.1293 [M–H]⁻), 6-C-α-L-arabinopyranosyl-8-*C*-*β*-D-xylopyranosyl-apigenin (**15**; RT = 4.70 min; *m/z* 533.1294 [M–H]⁻), 8-*C*-*α*-L-arabinopyranosyl-6-*C*-*β*-D-xylopyranosyl-apigenin (**16**; RT = 4.87 min; *m/z* 533.1292 [M–H]⁻), and apigenin 6,8-di-*C*-*α*-L-arabinopyranoside (**17**; RT = 4.95 min; *m/z* 533.1297 [M–H]⁻) were additionally detected. All flavones exhibited accurate masses typical of *C*-glycosylflavones and exhibited closely related elution times (3.8–5.0 min), suggesting structural similarity and isomerism within the apigenin derivatives. These compounds are commonly reported in Lamiaceae plants and are known for their antioxidant and anti-inflammatory activities.

***Phenolic compounds***

Several phenolic metabolites were identified, including both simple phenolic acids and phenylethanoid derivatives. Galloylglycerol (**5**; RT = 2.79 min; *m/z* 243.0498 [M–H]⁻), kelampayoside A (**6**; RT = 3.21 min; *m/z* 477.1609 [M–H]⁻), and melilotoside (**7**; RT = 3.39 min; *m/z* 325.0913 [M–H]⁻) were found as early-eluting phenolics with moderate polarity. Later-eluting phenolics such as rosmarinic acid (**24**; RT = 9.21 min; *m/z* 359.0763 [M–H]⁻) and dihydrocapsiate (**25**; RT = 9.40 min; *m/z* 307.1905 [M–H]⁻) were detected as caffeic- and vanillyl-type derivatives. A high-molecular-weight phenolic polyol, 1,2,3-trideoxy-4,6:5,7-bis-O-[(4-propylphenyl)methylene]nonitol (**41**; RT = 15.07 min; *m/z* 483.2728 [M–H]⁻), was also observed, reflecting phenolic condensation products. Overall, the phenolic profile indicates the coexistence of simple hydroxybenzoic acids and complex phenylethanoid structures.

***Monoterpenes and phthalates***

Three monoterpenoid and ester-like compounds were identified. Tuberonic acid glucoside (**8**; RT = 3.70 min; *m/z* 387.1647 [M–H]⁻) was found as a glycosylated oxylipin derivative. Two additional semi-volatile compounds were characterized as 1-(4-methyl-7-oxooctyl) 1,2-cyclohexanedicarboxylate (**26**; RT = 9.85 min; *m/z* 311.1853 [M–H]⁻) and dipentyl phthalate (**27**; RT = 10.07 min; *m/z* 305.1748 [M–H]⁻). Their retention behavior suggests relatively low polarity compared to the phenolic compounds.

***Glycosides and sugar-related metabolites***

Two sugar-containing compounds were identified. Nonioside B (**18**; RT = 5.54 min; m/z 629.2663 [M–H]⁻) was detected as a polyhydroxylated glycoside, while tetradecyl diglucosyl glyceride (**36**; RT = 13.58 min; m/z 699.3810 [M–H]⁻) was assigned as a long-chain glycosylated glyceride. These compounds indicate the presence of secondary glycosidic conjugates in the extract.

***Fatty acids and esters***

A large group of fatty acids and their esters were identified, reflecting the lipid-rich nature of Premna odorata extract. Azelaic acid (**19**; RT = 5.71 min; m/z 187.0966 [M–H]⁻), (9S,10E,12S,13S,15Z)-9,12,13-trihydroxy-10,15-octadecadienoic acid (**20**; RT = 7.83 min; m/z 327.2167 [M–H]⁻), pinellic acid (**21**; RT = 8.35 min; m/z 329.2321 [M–H]⁻), 10,16-dihydroxyhexadecanoic acid (**22**; RT = 8.62 min; m/z 287.2215 [M–H]⁻), and decanediol dimethacrylate (**23**; RT = 8.94 min; m/z 309.2068 [M–H]⁻) were the first series of aliphatic acids and esters detected. Several oxygenated linolenic acid derivatives were also identified, including (9Z,11E,15Z)-13-hydroxy-9,11,15-octadecatrienoic acid (**28**; RT = 12.15 min; m/z 293.2112 [M–H]⁻), (13S,9Z,11E,15Z)-13-hydroxy-9,11,15-octadecatrienoic acid (**29**; RT = 12.23 min; m/z 293.2115 [M–H]⁻), 17-hydroxylinolenic acid (**30**; RT = 12.28 min; m/z 293.2111 [M–H]⁻), 13-oxo-9Z,11E-octadecadienoic acid (**31**; RT = 12.33 min; m/z 293.2110 [M–H]⁻), 9-oxo-10E,12Z-octadecadienoic acid (**32**; RT = 12.42 min; m/z 293.2119 [M–H]⁻),
licanic acid (**33**; RT = 12.67 min; m/z 291.1963 [M–H]⁻), 13-hydroxy-9,11-octadecadienoic acid (**34**; RT = 13.18 min; m/z 295.2267 [M–H]⁻), and (±)-coriolic acid (**35**; RT = 13.27 min; m/z 295.2271 [M–H]⁻). These compounds showed sequential retention order consistent with the number of unsaturations and hydroxyl substituents. Additionally, ricinoleic acid (**38**; RT = 14.18 min; m/z 297.2425 [M–H]⁻) and several long-chain fatty acid esters were detected in the late chromatographic region, including methyl 12-hydroxystearate (**42**; RT = 17.17 min; m/z 313.2739 [M–H]⁻), heptadecanoic acid 2,3-dihydroxypropyl ester (**43**; RT = 17.28 min; m/z 343.2843 [M–H]⁻), cetyl lactate (**45**; RT = 17.81 min; m/z 313.2742 [M–H]⁻), 5-[(2-hydroxyethyl)amino]pentyl (9Z)-9-octadecenoate (**46**; RT = 17.87 min; m/z 410.3632 [M–H]⁻),
and glycol monostearate (**47**; RT = 18.14 min; m/z 327.2892 [M–H]⁻). These lipidic metabolites, characterized by long carbon chains and oxygenated moieties, are considered to contribute to membrane-related biological activities and anti-inflammatory effects.

***Triterpenes and triterpene lactones***

Several high-molecular-weight triterpenoid compounds were identified as secondary metabolites. Ganoderic acid H (**37**; RT = 13.87 min; m/z 571.2885 [M–H]⁻), 3-*O*-methyl-22*β*,23-dihydroxy-6-oxotingenol (**39**; RT = 14.45 min; m/z 481.2565 [M–H]⁻), Picrasinoside F (**40**; RT = 14.63 min; m/z 555.2834 [M–H]⁻), Mogroside I-E1 (**44**; RT = 17.70 min; m/z 637.4313 [M–H]⁻), and Cyclounifolioside C (**49**; RT = 18.44 min; m/z 653.4255 [M–H]⁻) were tentatively assigned. These triterpenoids, characterized by oxygenated rings and glycosidic linkages, are typical for *Premna* species and are known to exhibit anti-inflammatory, hepatoprotective, and cytoprotective properties.

***Miscellaneous metabolites***

Two minor metabolites of distinct structural classes were also identified. Kadsurenone (**48**; RT = 18.26 min; m/z 355.1573 [M–H]⁻) was characterized as a benzofuran derivative previously reported from Lauraceae plants, and 21-(acetyloxy)-17-hydroxypregna-1,4,9(11)-triene-3,20-dione (**50**; RT = 19.68 min; m/z 383.1886 [M–H]⁻) was identified as a steroidal metabolite. These compounds, although minor in abundance, may contribute to the bioactivity profile of the extract through synergistic effects with the major flavonoids and triterpenoids.

Based on the relative abundance observed in the PDA chromatograms at 254 nm (Figure 7B), flavonoids were identified as the predominant group of metabolites. Among them, compounds **11** (Area % = 5.32), **14** (Area % = 11.78), and **17** (Area % = 12.80) exhibited the highest relative abundances. These were followed by phenolic compounds **6** (Area % = 5.29), **7** (Area % = 4.32), and **24** (Area % = 4.82), which were present at intermediate levels. In contrast, other detected metabolites, such as fatty acids, carboxylic acids, glycosides, and triterpenoids, were found in comparatively low concentrations.

**Supplement materials and methods**

**Reagents and antibodies**

EZ-CYTOX (EZ-500) was purchased from DoGenBio (Seoul, Korea). Penicillin–streptomycin, Fetal bovine serum (FBS), Roswell Park Memorial Institute (RPMI) 1640, and Dulbecco's Modified Eagle medium (DMEM) were purchased from GenDEPOT (Baker, TX, USA). Ultrapure Lipopolysaccharide from P. gingivalis (LPS; tlrl-ppglps), flagellin (FLA-ST; tlrl-epstfla), ATP, and nigericin (tlrl-nig) were purchased from InvivoGen (San Diego, CA, USA). Highly cross-adsorbed mouse IgG (H + L) secondary antibodies (A32723), MitoProbe™ JC-1 Assay Kit (M34152), and Lipofectamine 2000™ (L3000015) were purchased from Invitrogen (Thermo Fisher Scientific, Waltham, MA, USA). Antibodies against ASC (sc-514414) and α-tubulin (sc-5286) were purchased from Santa Cruz Biotechnology (Santa Cruz, CA, USA), and an antibody against lamin A/C (A19664) was purchased from ABclonal (Woburn, MA, USA). Mouse TNF-alpha DuoSet ELISA (DY410) and Human IL-1β/ mouse IL-18 DuoSet ELISA (DY318-05) were purchased from R&D Systems. An antibody against Caspase-1 (p20) (mouse), mAb (Casper-1) (AG-20B-0042) was purchased from AdipoGen Life Sciences (San Diego, CA, USA). The pGL4.32 Luciferase Reporter Vector (E8491), Bright-Glo™ Luciferase Assay System (E2610), and ADP-Glo Kinase Assay (V6930) were purchased from Promega (Madison, WI, USA). Antibody against NF-κB p65 (8242) was purchased from Cell Signaling Technology (Danvers, MA, USA). The NE-PER™ nuclear and cytoplasmic extraction reagents (78833) were purchased from Thermo Fisher Scientific. Imiquimod (IMQ; HY-B0180) and phorbol 12-myristate 13-acetate (PMA; HY-18739) were purchased from Med Chem Express (South Brunswick Township, NJ, USA). ROS Assay Kit (DCFDA/H2DCFDA) and nuclei stained with mounting medium (DAPI) (ab104139) and an antibody against Gasdermin D (GSDMD) (ab209845) were purchased from Abcam (ab113851; Cambridge, UK). Disuccinimidyl suberate (DSS; 68-528-80-3) and N-acetyl-L-cysteine (NAC; A9165-25G) were purchased from Sigma-Aldrich (St Louis, MO, USA). Lipidofect-P (LDL-P001) was purchased from Lipidomia (Seoul, Korea).

***WST* assay**

Differentiated THP-1 cells and J774A.1 cells were seeded at (0.5 × 10⁵ cells/well) in 96-well plates for cytotoxicity assays. THP-1 cells were treated with POE (10, 50, or 100 µg/mL) and incubated overnight, while J774A.1 cells were treated with POE (10, 50, or 100 µg/mL) for 2 h. EZ-CYTOX was added for 2 h to assess cell viability. Absorbance was measured at 450 nm using an iMark™ microplate absorbance reader (Bio-Rad, 168-1130).

**Extraction of nuclear and cytoplasmic proteins**

J774A.1 cells (4.2 × 10⁶ cells) were treated with LPS (100 ng/mL) for 3 h and subsequently with POE (50 or 100 µg/mL) for 2 h. Cytoplasmic and nuclear fractions were isolated using the NE-PER™ Nuclear and Cytoplasmic Extraction Reagents (Thermo Scientific, #78833) according to the manufacturer’s instructions.

**ASC oligomerization**

LPS-primed THP-1 or NLRP3 Knockout (KO) THP-1 macrophages (1.2 × 10⁶ cells/well) were treated with POE (10, 50, or 100 µg/mL) for 2 h. Subsequently, nigericin (10 µM) was added to activate the inflammasome. The cells were collected, washed with PBS, and resuspended in PBS containing DSS (25 mM), followed by incubation for 30 min. Protein expression was then analyzed by Western blotting.

**Measurement of intracellular ROS**

Mouse J774A.1 cells (0.2 × 10⁶ cells/well) were treated with LPS (100 ng/mL) for 3 h, followed by an additional 2 h treatment with POE (50 µg/mL). NAC was co-treated with LPS for a total of 5 h. After removing the culture medium, the cells were washed with PBS. MitoSOX was diluted in PBS to a final concentration of 5 µM and applied to the cells, followed by incubation at 37 °C for 10 min. The solution was then removed, the cells were washed again with PBS, and ATP (5 mM) was added for a 5 min incubation at 37 °C. After treatment, cells were visualized using a confocal laser-scanning microscope (Carl Zeiss, LSM 710).

**Mitochondrial membrane potential assessment**

J774A.1 cells were seeded at 0.2 × 10⁶ cells/well in 4-well plates. Cells were primed with LPS (100 ng/mL, 300 µL) for 3 h, followed by POE (50 µg/mL) treatment for 2 h without medium change. Cells were then washed with pre-warmed PBS and incubated with JC-1 dye (10 µM in serum-free DMEM, 200 µL) at 37°C for 10 min in the dark. After washing, cells were stimulated with ATP (5 mM, 200 µL) for 5 min at 37°C. Fluorescence was visualized using a confocal laser-scanning microscope (Carl Zeiss, LSM710). JC-1 monomers indicating mitochondrial depolarization were detected at the green channel, and JC-1 aggregates indicating mitochondrial polarization were detected at the red channel.

**ASC speck staining**

Mouse J774A.1 cells were seeded on 4-well glass-bottom plates, primed with LPS (100 ng/mL) for 3 h, and treated with POE (100 µg/mL) for 2 h. Inflammasome activation was induced by nigericin (10 µM, 1 h). Cells were washed, fixed with 4% paraformaldehyde, permeabilized with 0.5% Triton X-100, and blocked with 1% BSA in PBST. Cells were incubated overnight at 4 °C with a mouse anti-ASC antibody, followed by Alexa Fluor–conjugated secondary antibody. Nuclei were stained with DAPI, and ASC specks were visualized using a confocal laser-scanning microscope (Carl Zeiss, LSM 710).

**Measurement of NLRP3 ATPase activity**

The recombinant human NLRP3 protein (0.139 mg/mL) was incubated with POE (100 μg/mL) or vehicle control (DMSO) in ATPase reaction buffer (100 mM Tris-HCl, pH 7.8; 2.8 mM EDTA; 100 mM MgCl₂, 15 mM KCl; 660 mM NaCl) at 25 °C for 3 h. Subsequently, ultrapure ATP (1 mM) was added, and the mixture was further incubated at 37 °C for 40 min. ATP hydrolysis by NLRP3 was evaluated using the ADP-Glo™ Max Assay (Promega, Madison, USA) according to the manufacturer’s instructions. Luminescence was measured using a microplate reader (BMG LabTech FLUOstar OPTIMA).

**Sudan Black B staining**

To assess neutrophil response to LPS and POE, embryos at 3 dpf were treated with 4% paraformaldehyde (PFA) overnight at 4 °C and washed three times with PBS. Embryos were incubated in Sudan Black B solution diluted in 70% Ethanol for 1 h at room temperature in the dark, followed by sequential washes with PBS-Tween and 70% ethanol. To remove non-neutrophilic pigmentation, embryos were incubated in a depigmentation solution (KOH, H₂O₂), washed with PBS-T and stored in PBS at 4 °C. The caudal hematopoietic tissue (CHT) region was imaged using a Leica M250 C microscope.

***In vivo* imaging of transgenic zebrafish**

To assess macrophage response to LPS and POE, transgenic *Tg (mpeg1*: EGFP*)* zebrafish were crossed with WT zebrafish to obtain embryos, which were maintained in PTU. At 3 dpf, following treatment with POE and LPS, live embryos were anesthetized with tricaine and imaged in the CHT region using Olympus FV3000RS confocal microscope.

**UPLC–PDA-QTOF–MS conditions**

A Waters Xevo G2-S QTOF mass spectrometer (Waters Corp., Milford, MA, USA) coupled to an ACQUITY UPLC I-Class system, which includes a photodiode array (PDA) detector and an ACQUITY UPLC BEH C18 reversed-phase column (100 × 2.1 mm, 1.7 μm, Waters) were used. The mobile phase solvents were water containing 0.1% formic acid (FA) (*v*/*v*) (Solvent A) and acetonitrile containing 0.1% FA (*v*/*v*) (Solvent B). The analytical gradient elution conditions were as follows: B 5% (0 min - 1 min) → 100% (20 min - 22.3 min) → 5% (22.4 min - 25 min). The flow rate was 0.4 mL/min, the column temperature was 35 °C, and the sample injection volume was 2 μL. QTOF–MS analysis was performed using electrospray ionization (ESI) with an ion source temperature of 110 °C and a desolvation temperature of 350 °C. Additionally, the capillary voltage was 2.3 kV, the cone voltage was 40 V, and the desolvation and cone gas flow rates were 800 L/h and 50 L/h, respectively. Accurate mass measurements were obtained with an automatic calibrated delivery system using the internal standard leucine-enkephalin [*m/z* 554.2615 (ESI-) and *m/z* 556.2771 (ESI+)]. The mass scan range was *m/z* 100-1500. All MS data were analyzed in MS^E^ mode and acquired using MassLynx (Waters) software.

**Molecular Docking**

*In silico* molecular docking was performed to assess the potential binding interactions between the major flavone constituents of POE and the NLRP3 NACHT domain. The crystal structure of human NLRP3 NACHT domain (PDB: 7ALV) was retrieved from the RCSB Protein Data Bank (<https://www.rcsb.org>) and used as the receptor. The co-crystallized ligand and water molecules were removed prior to docking. Molecular docking was performed using AutoDock Vina v1.2.7 with the following parameters: grid box center coordinates X = 14.17 Å, Y = 33.87 Å, Z = 133.71 Å; exhaustiveness = 32. Residues predicted to form hydrogen bond interactions with each ligand within 3.5 Å were identified and visualized using PyMOL v2.0 (Schrödinger, LLC, New York, NY).

**References**

Kim, K.H., Kim, Y.H., Lee, K.R., 2007. Isolation of quinic acid derivatives and flavonoids from the aerial parts of *Lactuca indica* L. and their hepatoprotective activity in vitro. Bioorg. Med. Chem. Lett. 17, 6739–6743. <https://doi.org/10.1016/j.bmcl.2007.10.046>

Takeuchi, Y., Nagao, Y., Toma, K., Yoshikawa, Y., Akiyama, T., Nishioka, H., Abe, H., Harayama, T., Yamamoto, S., 1999. Synthesis and siderophore activity of vibrioferrin and one of its diastereomeric isomers. Chem. Pharm. Bull. 47, 1284–1287. <https://doi.org/10.1248/cpb.47.1284>

Reva, I.D., Jarmelo, S., Lapinski, L., Fausto, R., 2004. IR-Induced photoisomerization of glycolic acid isolated in low-temperature inert matrices. *J. Phys. Chem. A* 108, 5036–5042. <https://doi.org/10.1021/jp0483627>

Kim, E.K., Kim, J.H., Jeong, S., Choi, Y.W., Choi, H.J., Kim, C.Y., Kim, Y.-M., 2019. Pachypodol, a Methoxyflavonoid Isolated from *Pogostemon cablin* Benth. Exerts Antioxidant and Cytoprotective Effects in HepG2 Cells: Possible Role of ERK-Dependent Nrf2 Activation. *Int. J. Mol. Sci.* 20, 4082. <https://doi.org/10.3390/ijms20174082>

Zhang, S., Akoh, C.C., 2020. Antioxidant property and characterization data of 1-o-galloylglycerol synthesized via enzymatic glycerolysis. *Data Brief.* 29, 105110. <https://doi.org/10.1016/j.dib.2020.105110>

Kitagawa, I., Wei, H., Nagao, S., Mahmud, T., Hori, K., Kobayashi, M., Uji, T., Shibuya, H., 1996. Indonesian Medicinal Plants. XIV. Characterization of 3′-O-Caffeoylsweroside, a new secoiridoid glucoside, and kelampayosides A and B, two new phenolic apioglucosides, from the bark of *Anthocephalus chinensis* (Rubiaceae). *Chem. Pharm. Bull.* 44, 1162–1167. <https://doi.org/10.1248/cpb.44.1162>

Afshar, F.H., Delazar, A., Nazemiyeh, H., Nahar, L., Moghaddam, S.B., Mbaebie, B.O., Gibbons, S., Sarker, S.D., 2017. Melilotoside derivatives from *Artemisia splendens* (Asteraceae). *Rec. Nat. Prod.* 11(1), 43–50. <https://doi.org/10.25135/rnp.11.16.09.325>

Wakuta, S., Hamada, S., Ito, H., Matsuura, H., Nabeta, K., Matsui, H., 2010. Identification of a *β*-glucosidase hydrolyzing tuberonic acid glucoside in rice (*Oryza sativa* L.). *Phytochemistry* 71(11-12), 1280–1288. <https://doi.org/10.1016/j.phytochem.2010.04.025>

Nguyen, H.S., Duong Thi, T.T., Nguyen Thi, K.T., Huynh Thi, H.T., Dao, D.T., 2025. Chemical constituents in the seeds of *Premna odorata*. *Lett. Org. Chem.* 22(9), 717–722. <https://doi.org/10.2174/0115701786360982250217054234>

Elmaidomy, A.H., Mohammed, R., Hassan, H.M., Owis, A.I., Rateb, M.E., Khanfar, M.A., Krischke, M., Mueller, M.J., Abdelmohsen, U.R., 2019. Metabolomic profiling and cytotoxic tetrahydrofurofuran lignans investigations from *Premna odorata* Blanco. *Metabolites* 9(10), 223. <https://doi.org/10.3390/metabo9100223>

Rayyan, S., Fossen, T., Andersen, Ø.M., 2010. Flavone C-glycosides from seeds of *Trigonella foenum-graecum* L. *J. Agric. Food Chem.* 58 (12), 7211–7217. https://doi.org/10.1021/jf100848c

Rauter, A.P., Lopes, R.G., Martins, A., 2007. C-Glycosylflavonoids: Identification, bioactivity and synthesis. *Nat. Prod. Commun.* 2(11), 1175–1196. <https://doi.org/10.1177/1934578X0700201125>

Nguyen, H.A., Do Thi, Y.H., Nguyen, T.C., Bui, H.T., Pham, H.Y., Pham, T.C., Pham, V.C., Nguyen, N.H., Kiem, P.V., Cho, S.-H., Park, S.J., Nguyen, X.N., 2023. Three new chromanes and one new flavone C-glycoside from *Mallotus apelta*. *J. Asian Nat. Prod. Res.* 25(1), 18–26. <https://doi.org/10.1080/10286020.2022.2051494>

Potterat, O., Von Felten, R., Dalsgaard, P.W., Hamburger, M., 2007. Identification of TLC markers and quantification by HPLC-MS of various constituents in noni fruit powder and commercial noni-derived products. *J. Agric. Food Chem.* 55(18), 7489–7494. https://doi.org/10.1021/jf071359a

Ayorinde, F.O., Osman, G., Shepard, R.L., Powers, F.T., 1988. Synthesis of azelaic acid and suberic acid from Vernonia galamensis seed oil. *J. Am. Oil Chem. Soc.* 65, 1774–1777. https://doi.org/10.1007/BF02542380

Hou, C.T., 1996. A novel compound, 12,13,17-trihydroxy-9(Z)-octadecenoic acid, from linoleic acid by a new microbial isolate *Clavibacter* sp. ALA2. *J. Am. Oil Chem. Soc.* 73, 1359–1362. https://doi.org/10.1007/BF02523497

Gaurav, S., Tiwari, K.N., Singh, J., 2024. Phytochemical and toxicological evaluation of aqueous leaf extract of Premna integrifolia L. in male BALB/c mice. *Phytomedicine Plus* 5(4), 100680. <https://doi.org/10.1016/j.phyplu.2024.100680>

Bakhsh, H.T., Abdelhafez, O.H., Elmaidomy, A.H., Aly, H.F., Younis, E.A., Alzubaidi, M.A., Algehainy, N.A., Altemani, F.H., Majrashi, M., Alsenani, F., Bringmann, G., Abdelmohsen, U.R., Mokhtar, F.A., 2024. Anti-Alzheimer potential of *Solanum lycopersicum* seeds: in vitro, in vivo, metabolomic, and computational investigations. *Beni-Suef Univ. J. Basic Appl. Sci.* 13, 1. https://doi.org/10.1186/s43088-023-00453-x

Liu, K., Tolley, H.D., Lee, M.L., 2012. Highly crosslinked polymeric monoliths for reversed-phase capillary liquid chromatography of small molecules. *J. Chromatogr. A* 1227, 96–104. https://doi.org/10.1016/j.chroma.2011.12.081

El Jezi, N., 2019. *Étude phytochimique et pharmacologique de l’espèce Centaurium erythraea Rafn subsp. erythraea (Gentianaceae)*. Thèse de doctorat, Université Clermont Auvergne, Clermont-Ferrand, France. <https://theses.hal.science/tel-03408808>

Dong, M., Oda, Y., Hirota, M., 2000. (10E,12Z,15Z)-9-hydroxy-10,12,15-octadecatrienoic acid methyl ester as an anti-inflammatory compound from *Ehretia dicksonii*. Biosci. Biotechnol. Biochem. 64(4), 882–886. https://doi.org/10.1271/bbb.64.882

Pohnert, G., Koch, T., Boland, W., 1999. Synthesis of volicitin: a novel three-component Wittig approach to chiral 17-hydroxylinolenic acid. *Chem. Commun.* 1999, 1087–1088. https://doi.org/10.1039/A902020I

Ko, Y.-C., Choi, H. S., Kim, S.-L., Lee, D.-S., 2023. 13-Oxo-9Z,11E-octadecadienoic acid down-regulates c-Myc and attenuates breast cancer cell stemness. *In Vivo* 37(3), 1085–1092. https://doi.org/10.21873/invivo.13183

Zhao, B., Tomoda, Y., Mizukami, H., Makino, T., 2015. 9-Oxo-(10E,12E)-octadecadienoic acid, a cytotoxic fatty acid ketodiene isolated from eggplant calyx, induces apoptosis in human ovarian cancer (HRA) cells. *J. Nat. Med.* 69(3), 296-302. https://doi.org/10.1007/s11418-015-0892-x

Spiteller, P., Spiteller, G., 1997. 9-Hydroxy-10,12-octadecadienoic acid (9-HODE) and 13-hydroxy-9,11-octadecadienoic acid (13-HODE): excellent markers for lipid peroxidation. *Chem. Phys. Lipids* 89 (2), 131-139. https://doi.org/10.1016/S0009-3084(97)00070-4

Sharma, S.K., Ali, M., Singh, H., Sultana, S., Mir, S.R., 2017. Phytochemical investigation of the roots of *Jatropha curcas* L. *Int. J. Adv. Pharm. Med. Bioallied Sci.* 5(2), 94–99.

Altyar, A.E., Ashour, M.L., Youssef, F.S., 2020. *Premna odorata*: Seasonal metabolic variation in the essential oil composition of its leaf and verification of its anti-ageing potential via in vitro assays and molecular modelling. *Biomolecules* 10(6), 879. https://doi.org/10.3390/biom10060879

Neilson, A.H., Hynning, P.Å., 1998. Complete ¹H- and ¹³C-NMR spectral assignments of salaciquinone, a new 7-oxo-quinonemethide dinortriterpenoid. *Tetrahedron* 54, 13579–13590. https://doi.org/10.1016/S0040-4020(98)00835-7

Song, X.-C., Canellas, E., Dreolin, N., Goshawk, J., Nerin, C., 2022. Identification of nonvolatile migrates from food contact materials using ion mobility-high-resolution mass spectrometry and in silico prediction tools. *J. Agric. Food Chem.* 70(30), 9499–9508. https://doi.org/10.1021/acs.jafc.2c03615

Wineburg, J.P., Swern, D., 1973. NMR chemical shift reagents in structural determination of lipid derivatives. III. Methyl ricinoleate and methyl 12-hydroxystearate. *J. Am. Oil Chem. Soc.* 50(5), 142–146. https://doi.org/10.1007/BF02640467

Ahmed, E.F., Sleem, A., Abbas, F.A., El-Shafae, A., 2020. Phytochemical constituents, HPLC-PDA-ESI-MS/MS profile and bioactivities of roots and rhizomes of *Prosopis farcta* (Banks & Sol.) J.F. Macbr. *Nat. Prod. J.* 10 (4), 411–428. https://doi.org/10.2174/2210315509666190618094434

Liu, H., Wang, C., Qi, X., Zou, J., Sun, Z., 2018. Antiglycation and antioxidant activities of mogroside extract from *Siraitia grosvenorii* (Swingle) fruits. *J. Food Sci. Technol.* 55(5), 1880–1888. https://doi.org/10.1007/s13197-018-3105-2

Unno, R., Matsutani, M., Matsumoto, N., Hirakawa, H., Shiwa, Y., Yoshikawa, H., Okamoto, S., 2020. Lactic acid bacterial diversity in Brie cheese focusing on salt concentration and pH of isolation medium and characterisation of halophilic and alkaliphilic lactic acid bacterial isolates. *Int. Dairy J.* 109, 104757. https://doi.org/10.1016/j.idairyj.2020.104757

Inventor, A., Inventor, B., 2024. Multivalent nucleic acid vaccines for coronavirus based on sequences derived from SARS-CoV-2 delta and omicron strains. WIPO Patent Application WO2024067861, filed 2024

Martin, J.B., Lutton, E.S., 1965. Preparation and phase behavior of positionally isomeric propylene glycol monoesters. *J. Am. Oil Chem. Soc.* 42(6), 529–533. https://doi.org/10.1007/BF02540095

Kim, K.H., Choi, J.W., Choi, S.U., Ha, S.K., Kim, S.Y., Park, H.J., Lee, K.R., 2011. The chemical constituents of *Piper kadsura* and their cytotoxic and anti-neuroinflammatory activities. *J. Enzyme Inhibition Med. Chem.* 26(2), 254–260. https://doi.org/10.3109/14756366.2010.496363

Cavina, G., Valvo, L., Gallinella, B., Porra, R., Savella, A.L., 1992. A rapid micro‐column HPLC method for the determination of drug impurities in pharmaceutical preparations. *J. Pharm. Biomed. Anal.* 10(6), 437–446. <https://doi.org/10.1016/0731-7085(92)80062-R>
